# Acute exposure to polystyrene nanoparticles promotes liver injury by inducing mitochondrial ROS-dependent necroptosis and augmenting macrophage-hepatocyte crosstalk

**DOI:** 10.1186/s12989-024-00578-6

**Published:** 2024-04-12

**Authors:** Junjie Fan, Li Liu, Yongling Lu, Qian Chen, Shijun Fan, Yongjun Yang, Yupeng Long, Xin Liu

**Affiliations:** 1 Department of Laboratory and Blood Transfusion of Jiangbei Campus, The First Affiliated Hospital of Army Medical University (The 958th hospital of Chinese People’s Liberation Army), 400000 Chongqing, China; 2 Medical Research Center, Southwest Hospital, Army Military Medical University, 400038 Chongqing, China

**Keywords:** Polystyrene nanoparticles, Liver injury, RAW 264.7 cells, Necroptosis, Mitochondrial ROS, Macrophage-hepatocyte crosstalk

## Abstract

**Background:**

The global use of plastic materials has undergone rapid expansion, resulting in the substantial generation of degraded and synthetic microplastics and nanoplastics (MNPs), which have the potential to impose significant environmental burdens and cause harmful effects on living organisms. Despite this, the detrimental impacts of MNPs exposure towards host cells and tissues have not been thoroughly characterized.

**Results:**

In the present study, we have elucidated a previously unidentified hepatotoxic effect of 20 nm synthetic polystyrene nanoparticles (PSNPs), rather than larger PS beads, by selectively inducing necroptosis in macrophages. Mechanistically, 20 nm PSNPs were rapidly internalized by macrophages and accumulated in the mitochondria, where they disrupted mitochondrial integrity, leading to heightened production of mitochondrial reactive oxygen species (mtROS). This elevated mtROS generation essentially triggered necroptosis in macrophages, resulting in enhanced crosstalk with hepatocytes, ultimately leading to hepatocyte damage. Additionally, it was demonstrated that PSNPs induced necroptosis and promoted acute liver injury in mice. This harmful effect was significantly mitigated by the administration of a necroptosis inhibitor or systemic depletion of macrophages prior to PSNPs injection.

**Conclusion:**

Collectively, our study suggests a profound toxicity of environmental PSNP exposure by triggering macrophage necroptosis, which in turn induces hepatotoxicity via intercellular crosstalk between macrophages and hepatocytes in the hepatic microenvironment.

**Supplementary Information:**

The online version contains supplementary material available at 10.1186/s12989-024-00578-6.

## Background

The rapid expansion of global plastic manufacturing and application, coupled with the discharge of millions of tons of plastic waste worldwide annually, has resulted in significant environmental burdens and posed substantial threats to human health [1]. Plastic materials are known for their limited biodegradability, showing the typical properties of poorly soluble particles of low toxicity (PSLT) materials [1]. However, they may be intentionally synthesized at smaller sizes or fragmented from macroplastic items into smaller particles, which are referred to as primary or secondary microplastics (MPs) (≤ 5 mm in diameter) and nanoplastics (NPs) (≤ 100 nm in diameter). Regardless of their origin, smaller plastic particles display an augmented capacity to penetrate biological barriers, leading to increased ingestion and deposition in major organs of living organisms that may impose potential hazardous consequences [2].

In comparison to MPs of the same materials, NPs are even smaller in size but demonstrate higher active surface physiochemical properties [3]. Consequently, NPs are more likely to traverse across cell membranes, disrupt intracellular homeostasis, and promote cellular injury [3, 4]. Indeed, a substantial body of recent literature has documented the cytotoxicity of NPs in aquatic organisms and mammalian systems following direct cellular contact or systemic ingestion, including epithelial cells in the route of NPs entry and immune cells involved in NP clearance [1, 5, 6]. Of particular note are findings that plastic particles may penetrate biological barriers and repeatedly appear in the blood circulation of humans after exposure through multiple entry routes, where most of them are nonspecifically internalized by circulatory monocytes and resident macrophages [7]. While this process is considered essential for mediating the sequestration and clearance of NPs, it has recently become evident that the uptake of NPs may significantly disrupt the structural integrity of organelles in monocytes and macrophages, leading to a range of functional abnormalities, including lysosomal dysfunction [8], aberrant lipid metabolism [9] and overactivated inflammasomes [10]. Despite these findings, the specific harmful impact of NPs on monocytes/macrophages and the underlying mechanisms driving the injury or cell death process are not fully understood. Furthermore, the potential consequences of NP-exposed macrophages and their associated cellular abnormalities on major tissues and organs remain to be further explored.

The liver is the principal metabolic organ for the clearance of environmental substances in mammals. Importantly, a large proportion of administered nanoparticles (approximately 30–99%) are sequestered in the liver, involving uptake and trafficking by hepatic macrophages [11]. Several recent studies have suggested that NPs are also preferentially deposited in the liver, where they may disrupt lipid and energy metabolism [4, 12]. However, most in vivo studies have primarily focused on detecting the distribution and accumulation of nanoparticles at the organ level [11]. Meanwhile, existing toxicology experiments have only evaluated the direct cytotoxicity of NPs without considering potential cell-to-cell interactions. In fact, the interplay between different tissue cells, particularly that which occurs between macrophages and hepatocytes, has become increasingly recognized as important for understanding the mechanisms of liver injury due to exposure to either biological compounds or materials at nanoscales [13].

Polystyrene (PS) is a widely used plastic polymer material for manufacturing containers for food and water, leading to the widespread environmental distribution of polystyrene micro (nano) plastics and high levels of exposure to living organisms [14]. Importantly, emerging evidence strongly implicates the harmful impact of their exposure [15, 16]. However, it is challenging to obtain and quantify environmentally degraded PS plastics in a standardized manner due to methodological limitations [17]. Instead, synthetic PS nanoparticles (PSNPs) or nanobeads are widely used to assess their potential toxic effects. Henceforth, in this study, we aimed to determine the exact route of the toxic effect induced by synthetic PS beads/particles in macrophages and explore the underlying mechanisms. Given the preferential distribution of PS in the liver, we also investigated the potential routes of PS exposure that involved modulation of macrophage-hepatocyte crosstalk.

## Results

### Characterization of PSMPs/PSNPs

**Fig. 1:**
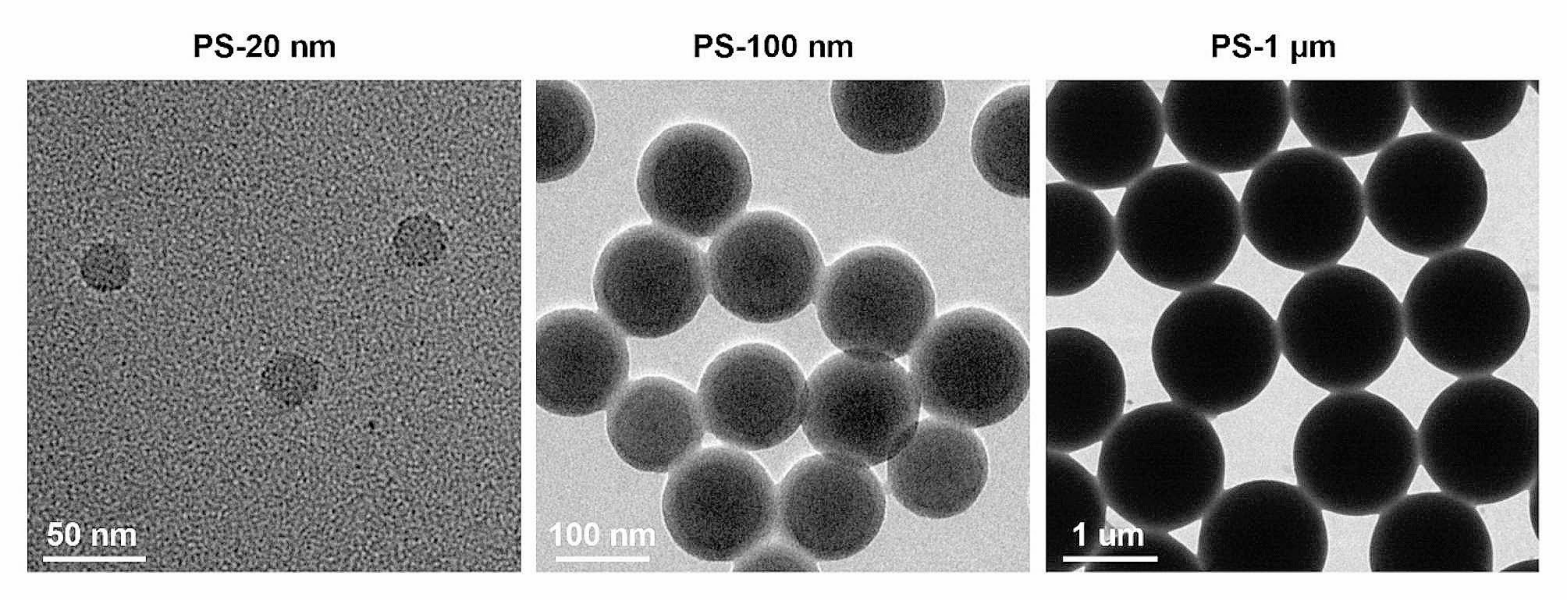
TEM images of 20 nm, 100 nm and 1 μm PS plastics

The morphology and characteristics of PSMPs (1 μm) and PSNPs (20 and 100 nm) were meticulously investigated using transmission electron microscopy (TEM), which revealed uniformly spherical particles, as depicted in (Fig. 1A). Subsequent analysis using a Zetasizer Nano ZS instrument provided insights into the average size distribution, polydispersity index (PDI) value, and zeta potential of the nanoparticles. Notably, all PSMPs/PSNPs exhibited a relatively low PDI (S. Table 1), indicative of a high degree of homogeneity within the particle population [18]. In both distilled water (DI) and the Dulbecco’s modified Eagle medium (DMEM), the average sizes of the 20 and 100 nm PSNPs closely matched their intended theoretical values. In contrast, the average diameter of the 1 μm PSMPs was significantly augmented (S. Figure 1A), it was potentially attributable to their heightened propensity for aggregation or the formation of a protein corona in the culture medium [19]. Furthermore, the absolute values of zeta potential for PSMPs/PSNPs were observed to be higher in deionized water than in DMEM, reflecting nonspecific binding with salt ions and charged biomolecules in the medium (S. Figure 1B).

### Enhanced cytotoxicity of smaller PSNPs in macrophages

**Fig. 2:**
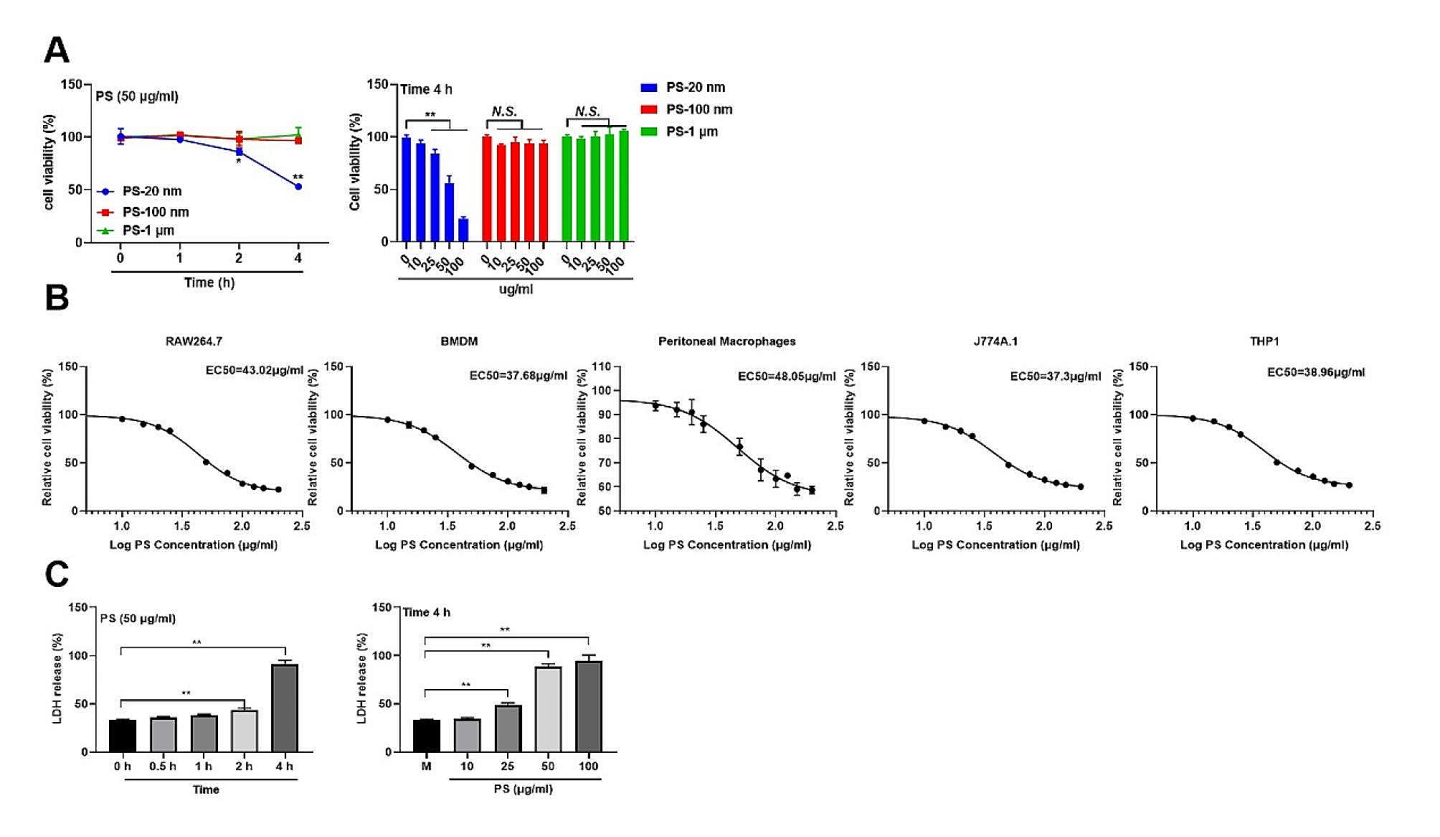
Identification of cytotoxic effects in macrophages induced by PSNPs. (**A**) Comparison of the time and dose effects on the viability of RAW 264.7 cells by 20 nm, 100 nm and 1 μm PS particles. Asterisks indicate significant differences from the control group. (**B**) Effect of 20 nm PSNPs (50 µg/mL) on the viability of RAW 264.7, BMDMs, peritoneal macrophages, J774A.1 cells and THP-1 cells after 4 h exposure. (**C**) LDH release in RAW 264.7 cells treated with 20 nm PSNPs at the indicated concentrations or for the indicated time. The results are presented as the mean ± S.D. *n* = 3, N.S., no significance. * P < 0.05, ** P < 0.01

**Fig. 3:**
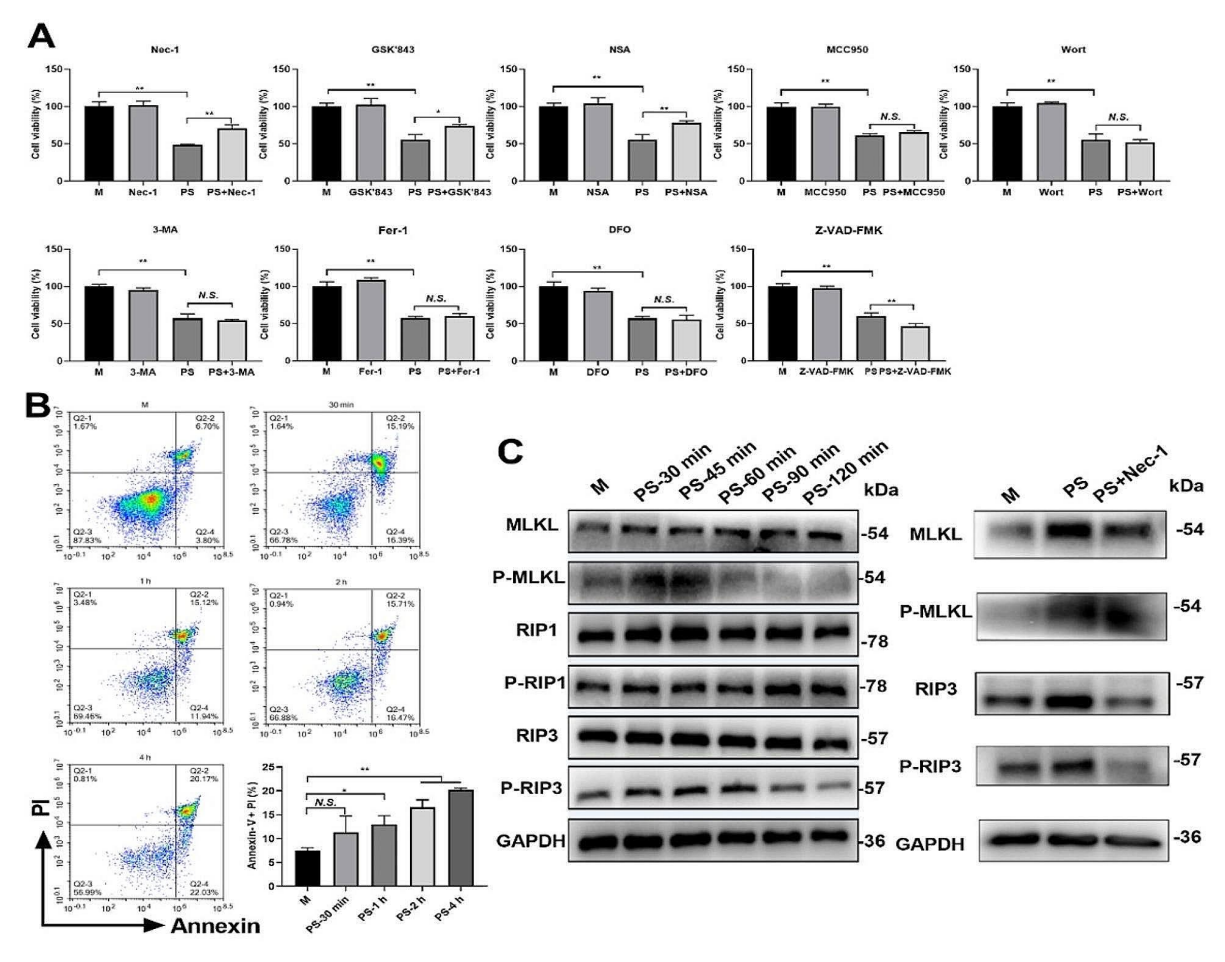
Necroptosis detection in RAW 264.7 cells after 20 nm PSNPs treatment. (**A**) The viability of RAW 264.7 cells after PSNPs treatment alone or together with necroptosis inhibitors Necrostatin-1 (Nec-1), GSK’843 and necrosulfonamide (NSA), pyroptosis inhibitors (MCC950), autophagy inhibitors (wortmannin (Wort) and 3-methyladenine (3-MA)), ferroptosis inhibitors (Ferrostatin-1 (Fer-1) and deferoxamine (DFO)) and apoptosis inhibitors (Z-VAD-FMK). (**B**) Late apoptosis/necroptosis in PSNPs treated RAW 264.7 cells at the indicated time. (**C**) The activation of necroptosis specific proteins in PSNPs-treated RAW 264.7 cells at the indicated time or together with the necroptosis inhibitor Nec-1 for 45 min. The concentration of PSNPs was 50 µg/mL unless otherwise indicated. The results are presented as the mean ± S.D. *n* = 3, N.S., no significance. * P < 0.05, ** P < 0.01

The cytotoxic effects of PSMPs/PSNPs on RAW 264.7 cells were meticulously investigated to discern their impact on cell viability. Intriguingly, it was discerned that the viability of RAW 264.7 cells was markedly diminished when exposed to a concentration exceeding 25 µg/mL of 20 nm PSNPs for 2–4 h, whereas the viability remained unaffected by the presence of 100 nm PSNPs or 1 μm PSMPs (Fig. 2A). This observation underscores the heightened toxicity of smaller PSNPs compared to larger particles. To further quantify the dose-toxicity of PSNPs, a series doses of PSNPs were given to RAW 264.7 cells and an EC50 value that reduced relative cell viability by 50% compared to the control, was 43.02 (40.82–45.36) µg/ml (with 95% confidence interval) (Fig. 2B). Furthermore, PSNPs were found to disrupt the viability of various macrophage cell lines, EC50 of BMDM, Peritoneal macrophages, J774A.1, THP1 cell lines was 37.68 (35.12 to 40.53) µg/ml, 48.05 (38.09 to 66.49) µg/ml, 37.3 (35.63 to 39.08) µg/ml, 38.96 (36.84 to 41.26) µg/ml, respectively (Fig. 2B). Underscoring their profound cytotoxic impact on macrophages or precursor monocytes. Consistent with these findings, the release of lactate dehydrogenase (LDH) from RAW 264.7 cells, indicative of membrane rupture and the severity of lytic cell death, was significantly elevated with increasing culture time and PSNPs concentration (Fig. 2C). Subsequently, it was established that PSNPs exerted a more pronounced effect on cell viability in a serum-free culture environment, potentially due to the interference of serum proteins with the interaction between PSNPs and the cell surface (S. Figure 2) Our cytotoxicity experiments showed that TiO2, as a PSLT, had no significant inhibitory effect on the viability of RAW 264.7 cells, but promoted cell proliferation (S. Figure 3). This further suggests the size and type of PSLT materials have different effects on cytotoxicity.

### PSNPs trigger necroptosis selectively in RAW 264.7 cells

To delineate the mode of cell death induced by PSNPs in RAW 264.7 cells, we employed a group of cell death inhibitors, including necroptosis inhibitors (Necrostatin-1, GSK’843 and necrosulfonamide), apoptosis inhibitors (Z-VAD-FMK), pyroptosis inhibitors (MCC950), autophagy inhibitors (wortmannin and 3-MA) and ferroptosis inhibitors (Ferrostatin-1 and deferoxamine). As shown in Fig. 3A, Necrostatin-1, GSK’843, and necrosulfonamide substantially restored the viability of RAW 264.7 cells that had been reduced by PSNPs treatment. However, the inhibition of autophagy, pyroptosis or ferroptosis did not attenuate the cytotoxicity of PSNPs. These findings suggest that PSNPs selectively induce necroptosis in macrophages. Intriguingly, co-treatment of RAW 264.7 cells with PSNPs and apoptosis inhibitors further reduced cell viability, which may support the notion that inhibition of apoptosis facilitates the transition into necroptosis. To confirm the direct induction of necroptosis by PSNPs, we examined the late apoptosis/necroptosis of RAW 264.7 cells using Annexin V-FITC/PI staining (Fig. 3B). As expected, we observed that PSNPs time-dependently increased the percentage of late apoptotic/necroptotic cells. Moreover, PSNPs treatment led to a rapid elevation of P-MLKL, P-RIP1 and P-RIP3 in RAW 264.7 cells (Fig. 3C), which was significantly disrupted by co-treatment with the necroptosis inhibitor Nec-1. Taken together, these results suggest that PSNPs directly induce necroptosis in macrophages.

### PSNPs induce necroptosis by augmenting mitochondrial ROS generation

**Fig. 4:**
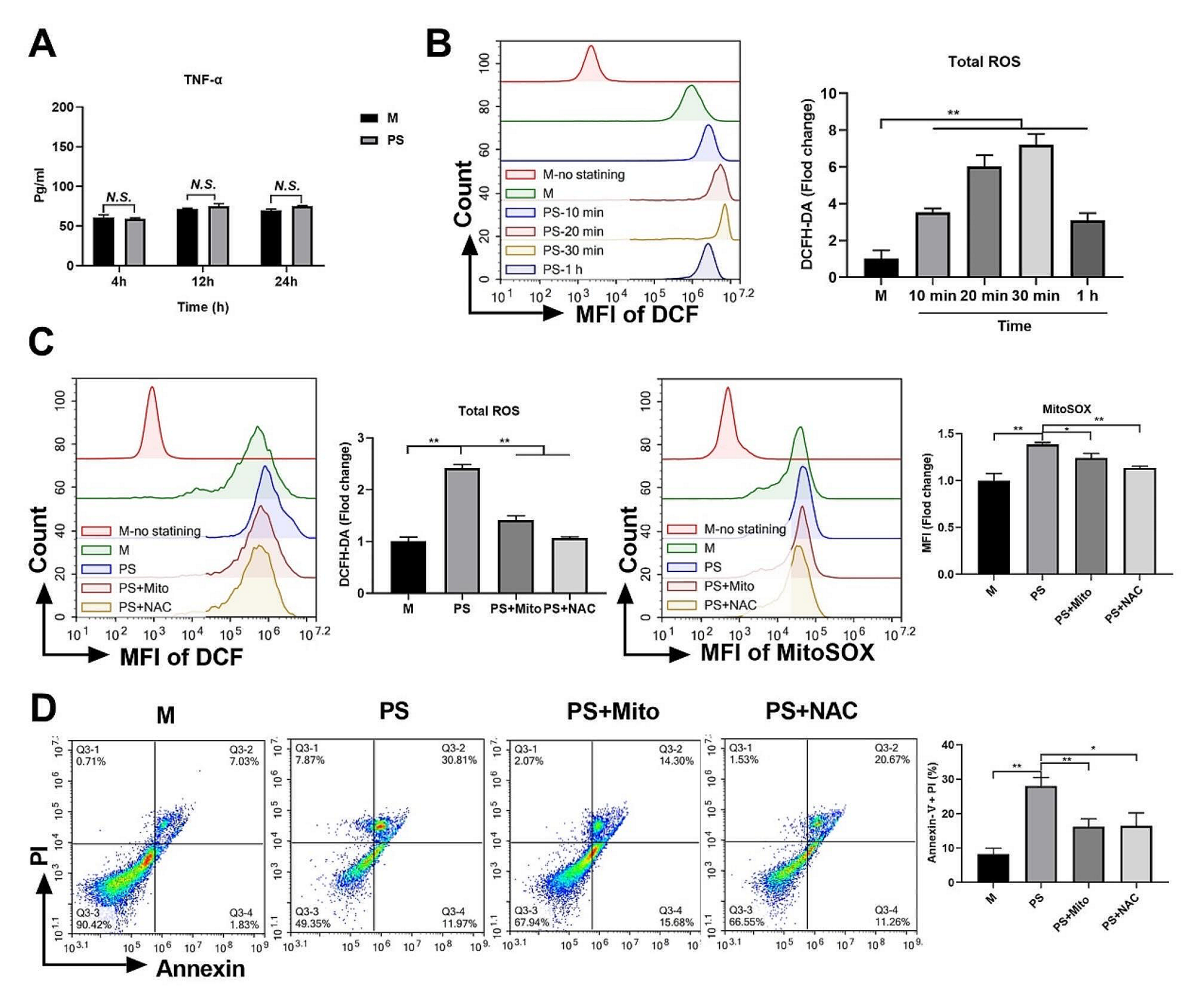
PSNPs treatment upregulates mitochondrial ROS generation to promote necroptosis in RAW 264.7 cells. (**A**) TNF-α quantification in RAW 264.7 cells treated with PSNPs for 4, 12, 24 h. (**B**) Total intracellular ROS levels in RAW 264.7 cells treated with 50 µg/mL PSNPs from 10 min to 1 h. (**C**) RAW 264.7 cells were treated with PSNPs with or without Mito-TEMPO or NAC for 30 min. The level of cytosolic ROS levels and mtROS were determined by dichlorodihydrofluorescein diacetate (DCFH-DA) staining and MitoSOX red staining, respectively. (**D**) Detection of late apoptotic/necroptotic cells in RAW 264.7 cells treated with or without Mito-TEMPO or NAC for 4 h. The results are presented as the mean ± S.D. *n* = 3, N.S., no significance. * P < 0.05, ** P < 0.01

ROS and TNF-α are both pivotal mechanisms that drive necroptosis in cells [20]. We found that PSNPs rapidly increased the production of ROS in RAW 264.7 cells but did not increase the production of TNF-α even after 24 h of exposure (Fig. 4A, B). These findings suggest that ROS, rather than TNF-α, may be the primary driver of necroptosis in PSNPs-treated macrophages. To further investigate the role of ROS in PSNPs-induced necroptosis, we examined a series of oxidative stress-related redox indicators, including superoxide dismutase (SOD), total mercapto (-SH), glutathione (GSH), and malondialdehyde (MDA), in RAW 264.7 cells after PSNPs exposure. We found that PSNPs significantly downregulated the activities of antioxidant SOD, -SH and GSH, while significantly upregulating the levels of the oxidation product MDA in a dose-dependent manner (S. Figure 4). Furthermore, we used a ROS scavenger N-acety-L-cysteine (NAC) and a mitochondrial targeting antioxidant (Mito-TEMPO) to distinguish the source of ROS and to identify the ROS-dependent mechanisms by which PSNPs promote necroptosis in RAW 264.7 cells. Both NAC and Mito-TEMPO reduced the increase of intracellular ROS and mtROS in PSNPs-treated RAW 264.7 cells, and also downregulated the percentage of necroptotic cells (Fig. 4C, D). These results suggest that PSNPs promote macrophage necroptosis by triggering the generation of mtROS.

### PSNPs are internalized by macrophages and accumulate in mitochondria

**Fig. 5:**
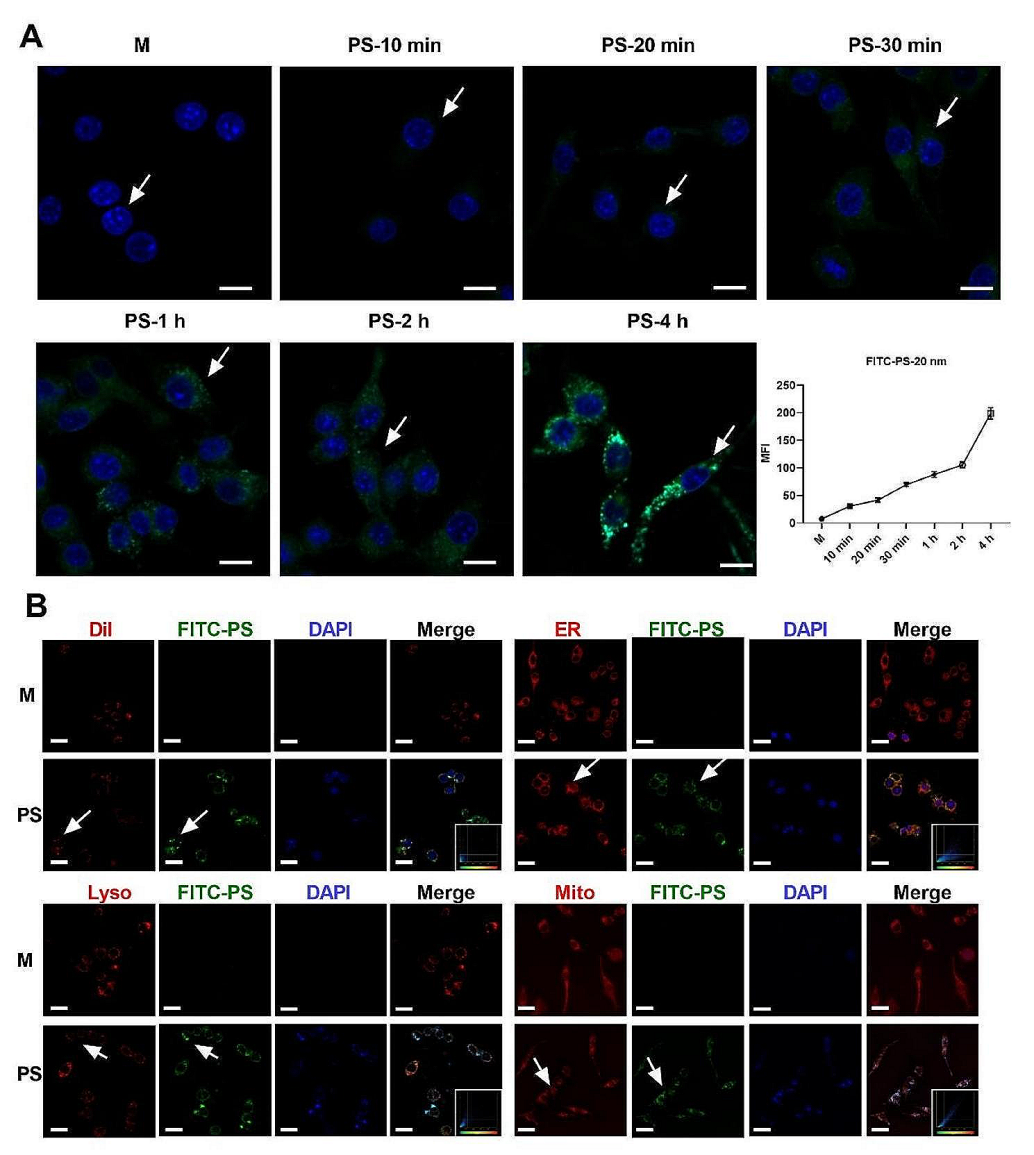
PSNPs uptake and suborganelle localization. (**A**) Time dependent uptake of PSNPs by RAW 264.7 cells. Scale bar 20 μm. (**B**) Overlap of FITC labeled PSNPs (fPSNPs) with the cell membrane (Dil), endoplasmic reticulum (ER), lysosome (Lyso) and mitochondria (Mito) in RAW 264.7 cells. Colocalization was calculated by the Pearson correlation coefficient. Scale bar 20 μm

**Fig. 6:**
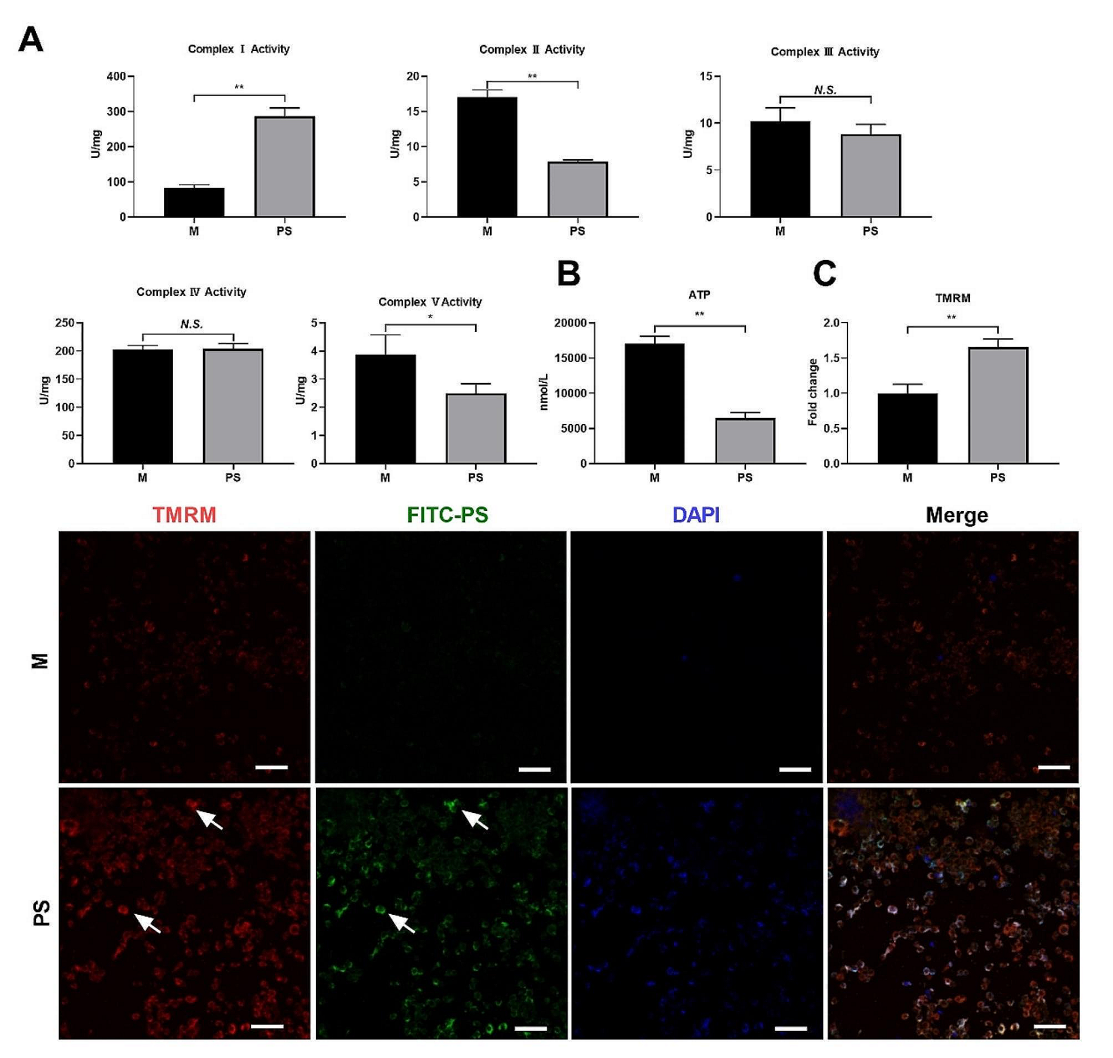
PSNPs disrupt the integrity and function of mitochondria in RAW 264.7 cells. (**A**) The activity of mitochondrial complexes I-V in RAW 264.7 cells treated with or without PSNPs for 4 h. (**B**) ATP production in RAW 264.7 cells treated with PSNPs for 4 h. (**C**) Detection of the cell mitochondrial membrane potential after PSNPs treatment for 4 h. Scale bar 50 μm. N.S., no significance. * P < 0.05, ** P < 0.01

**Fig. 7:**
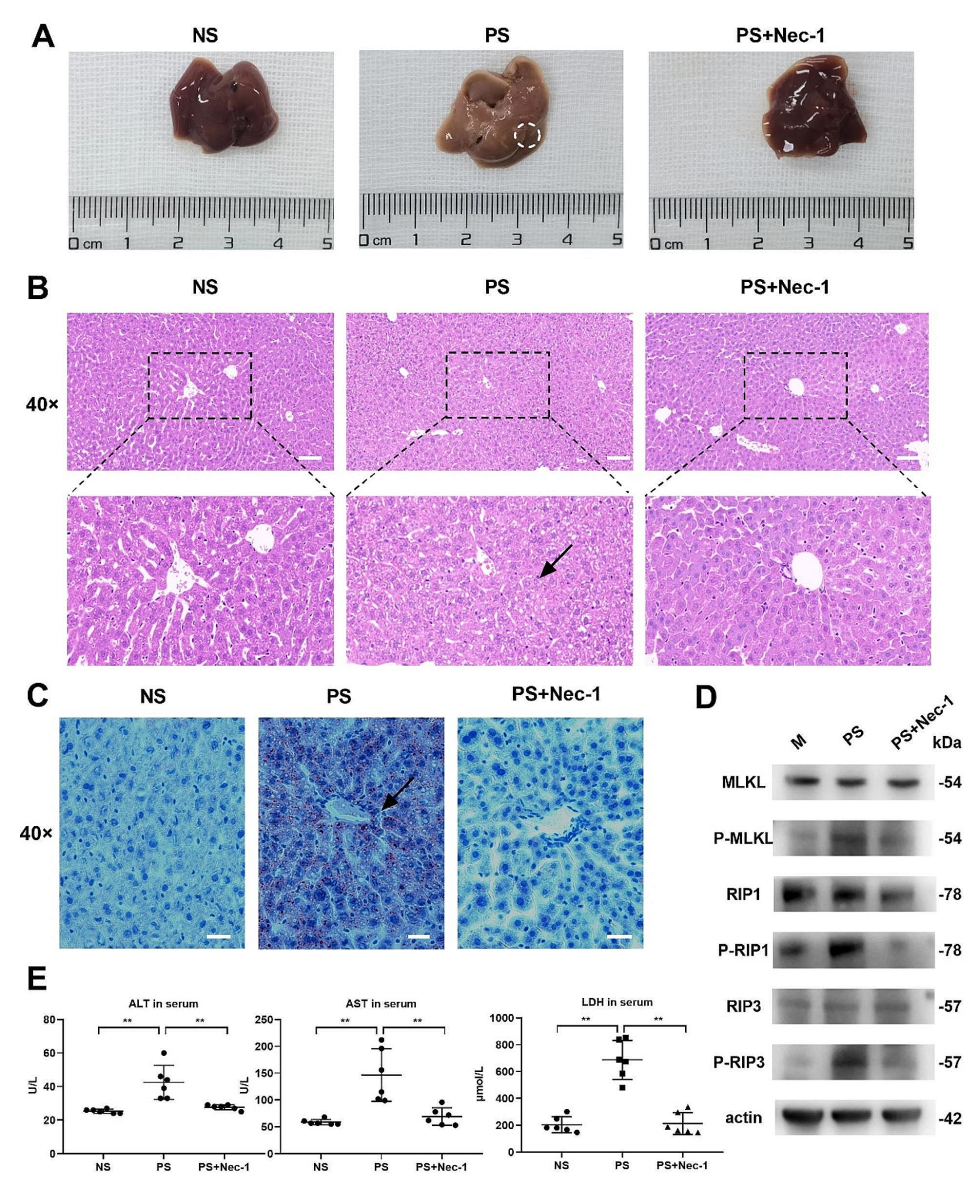
Enhancement of murine hepatic necroptosis and injury after PSNPs injection. BALB/c mice were injected with 60 mg/kg PSNPs alone or together with 50 µM Nec-1. (**A**) Morphological images of liver tissues. (**B**) Representative histopathological micrographs of liver sections in mice examined via H&E staining. Scale bar, 50 μm. (**C**) The severity of hepatic steatosis was determined via Oil red O staining, as indicated by red lipid droplets in hepatocytes. Scale bar, 50 μm. (**D**) Western blot analysis of necroptosis proteins in liver tissue. (**E**) Serum ALT, AST and LDH levels. Samples were obtained 24 h post-challenge (*n* = 6). N.S., no significance. * P < 0.05, ** P < 0.01

The internalization efficiency and subcellular distribution of PSNPs may account for their cytotoxicity. To investigate this, we exposed RAW 264.7 cells to FITC-labeled PSNPs (fPSNPs) for up to 4 h, and evaluated their internalization by LSCM and flow cytometry assays. We found that fPSNPs could be internalized into RAW 264.7 cells in a time-dependent manner, as indicated by the stronger fluorescence intensity with increasing incubation time as detected by confocal images (Fig. 5A) and flow cytometry (S. Figure 5). We also compared the subcellular localization of fPSNPs in RAW 264.7 cells treated with the same concentration of PSNPs for 4 h. We observed a significant overlap of fPSNPs with MitoBright LT Red (mitochondria) and ER-Tracker Red (endoplasmic reticulum), but far less overlap with Dil (cell membrane) and lyso-Tracker Red (lysosome) (Fig. 5B). The Pearson correlation coefficient (Rr) was calculated to quantify the subcellular organelle colocalization [21], and the Rr values of fPSNPs with mitochondria, endoplasmic reticulum, lysosome, and cell membrane in RAW 264.7 cells were 0.87 ± 0.02, 0.74 ± 0.04, 0 ± 0.28, and − 0.05 ± 0.32, respectively. To further verify the mitochondrial location of PSNPs, we treated J774A.1 cells with Nile Red-labeled PS. We consistently observed that these particles also accumulated in mitochondria as indicated by S. Figure 6. These results suggest that PSNPs may preferentially accumulate in the mitochondria and endoplasmic reticulum of RAW 264.7 cells. By using TEM, we also observed the structural changes of mitochondria in RAW264.7 cells After PS treatment, the mitochondrial cristae were fuzzied and the mitochondrial structure was fractured (S. Figure 7).

### PSNPs promote mitochondrial dysfunction to induce mtROS production

As PSNPs were found to accumulate in the mitochondria of RAW 264.7 cells, we investigated whether they induced mitochondrial dysfunction, leading to upregulated mtROS generation and subsequent necroptosis. We found that PSNPs increased the activity of complex I on the electron transport chain but reduced the activity of complex II and complex V (Fig. 6A). This was accompanied with significantly downregulated intracellular ATP levels (Fig. 6B) and a markedly increased mitochondrial membrane potential (MMP) in RAW 264.7 cells (Fig. 6C). These results suggest that PSNPs disrupt mitochondrial homeostasis, thereby inhibiting ATP generation while promoting ROS generation.

### PSNPs trigger hepatic necroptosis and induce hepatic injuries in mice

**Fig. 8:**
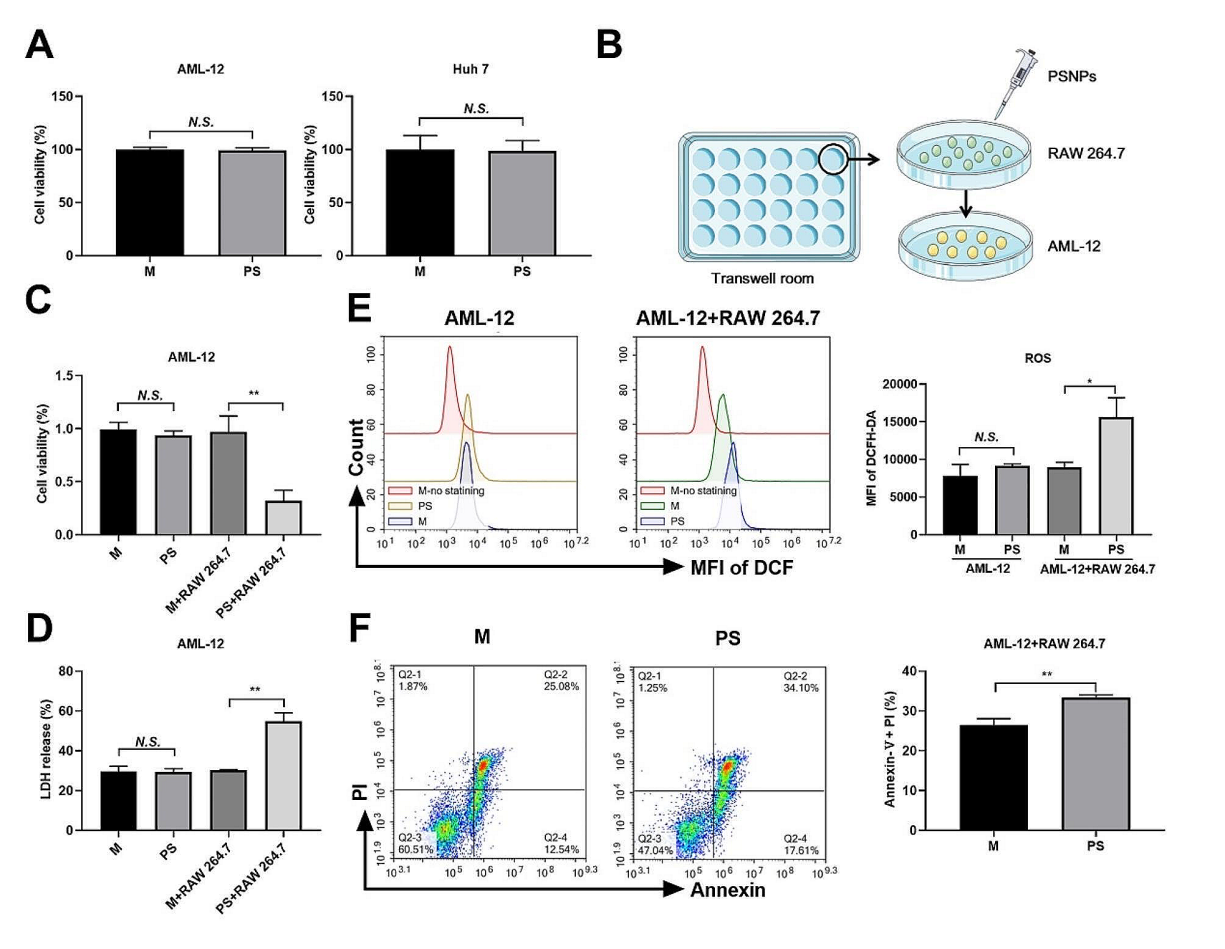
PSNPs induce macrophage-hepatocyte crosstalk to promote cell death in hepatocytes. (**A**) Toxic effect of PSNPs on AML-12 and Huh7 cells. (**B**) AML-12 and RAW 264.7 cell coculture diagram. (**C**-**D**) Cell viability and LDH release were measured in independently cultured AML-12 cells or a coculture of AML-12 cells with RAW 264.7 cells in a transwell system. (**E**-**F**) Flow cytometry analysis of the levels of ROS and late apoptotic/necroptotic cells of AML-12 cells co-cultured with RAW 264.7 cells. *n* = 3. N.S., no significance. * P < 0.05, ** P < 0.01. 20 nm PSNPs at 50 µg/mL or 60 mg/kg were used in vitro or in vivo respectively

To investigate whether the necroptotic cytotoxicity of PSNPs is responsible for tissue injuries, BALB/c mice were injected with PSNPs, and hepatic injuries were assessed. The histopathological examination demonstrated that PSNPs administration resulted in severe hepatic injury, including cell swelling, disorganized hepatic cord structure, and elevated infiltration of blood cells (Fig. 7A, B). Moreover, the pathology of hepatic damage caused by PSNPs was less pronounced by concomitant administration of Nec-1. Oil Red O (ORO) staining of liver sections also confirmed that the presence of hepatocyte steatosis, such as abnormally large lipid droplets and higher lipid accumulation, was upregulated post PSNPs treatment but reversed by Nec-1 (Fig. 7C). Additionally, the activation of necroptosis-associated signaling molecules, including P-MLKL, P-RIP1, and P-RIP3, was profoundly enhanced by PSNPs administration (Fig. 7D). Moreover, Nec-1 reduced the upregulation of P-MLKL, P-RIP1, and P-RIP3. In line with the histopathological observations, the levels of serum ALT, AST, LDH, and lipid peroxidation product MDA in the PSNP group were significantly upregulated, while the antioxidative stress index (SOD, -SH, and GSH) was downregulated (Fig. 7E, S. Figure 8). These indicators that reflect acute liver injury were significantly improved in the PSNP and Nec-1 co-injected group. Taken together, our results indicate that PSNPs induce necroptosis in live tissues and thus promote acute liver injury.

### PSNPs promote liver injury by enhancing the crosstalk between RAW 264.7 cells and AML-12 cells

The deleterious effects of nanoparticles on hepatocytes have been well documented [13]. Surprisingly, our investigation revealed that murine AML-12 hepatocytes and human Huh7 hepatocarcinoma cells were impervious to the toxic effects of PSNPs, thereby possibly excluding any direct hepatotoxicity (Fig. 8A). Instead, we postulated that PSNPs could promote liver injury by intensifying intercellular crosstalk in hepatic cells. To explore this possibility, RAW 264.7 cells and AML-12 cells were co-cultured in transwell chambers (Fig. 8B). Treatment of AML-12 cells with PSNPs alone in the chambers did not elicit any cytotoxicity, nor did it result in hepatocyte membrane rupture, LDH release, or ROS production. However, PSNPs treatment induced a decline in AML-12 cell viability, membrane rupture, and an increase in ROS generation when co-cultured with RAW 264.7 cells (Fig. 8C, D, E). Concomitantly, the proportion of necroptotic AML-12 cells was significantly higher when exposed to PSNPs in the presence of RAW 264.7 cells (Fig. 8F).

**Fig. 9:**
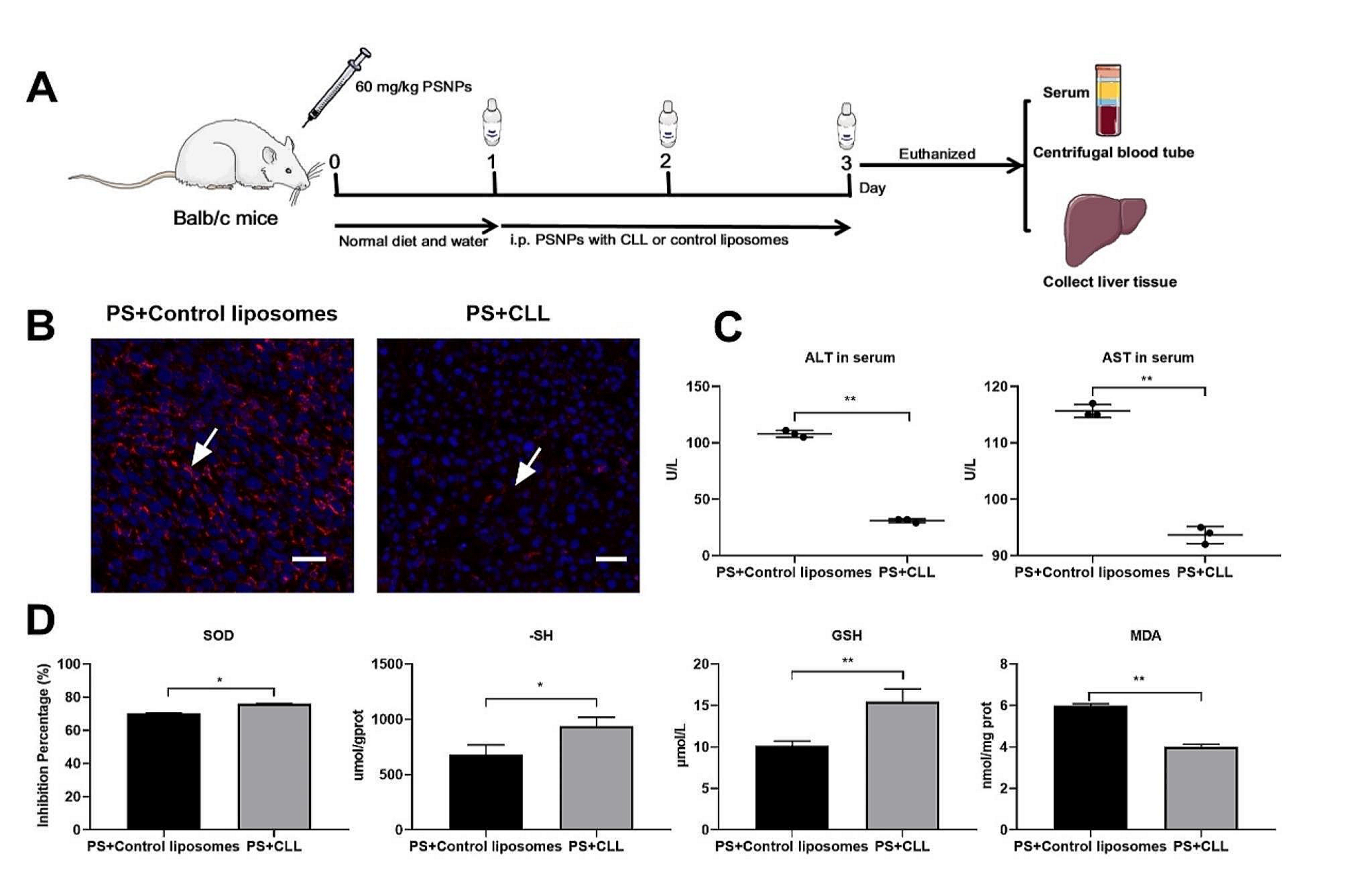
PSNPs induce macrophage-hepatocyte crosstalk cause liver injury. (**A**) BALB/c mice were preinjected with clodronate liposomes (CLLs) or control liposomes for 3 consecutive days and then challenged with PSNPs for 24 h. The depletion of macrophages was demonstrated by immunofluorescence staining of F4/80 and DAPI in frozen sections of mouse liver. Scale bar, 50 μm (**B**). Serum ALT and AST levels (**C**) and liver tissue oxidative damage levels of SOD, -SH, GSH and MDA (**D**) were detected. *n* = 3. *N.S.*, no significance. * *P* < 0.05, ** *P* < 0.01. 20 nm PSNPs at 50 µg/mL or 60 mg/kg were used in vitro or in vivo respectively

To further validate the role of hepatic macrophages in PSNPs-mediated liver injury, these cells were depleted in mice via repeated CLL injection (Fig. 9A). A pronounced decrease in F4/80 positive staining (a macrophage-specific marker) was detected in the liver tissues of mice after CLL injection and PSNPs challenge, denoting a widespread loss of hepatic macrophages (Fig. 9B). Furthermore, serum levels of ALT and AST, indices of liver injury, were considerably lower in macrophage-depleted mice compared to control mice following PSNPs administration (Fig. 9C). Correspondingly, liver injury markers, including SOD, -SH, GSH, and MDA, were substantially reversed in macrophage-depleted mice relative to control mice (Fig. 9D). Together, our results suggest that PSNPs may promote hepatic injury by enhancing the crosstalk between macrophages and hepatocytes.

## Discussion

**Fig. 10:**
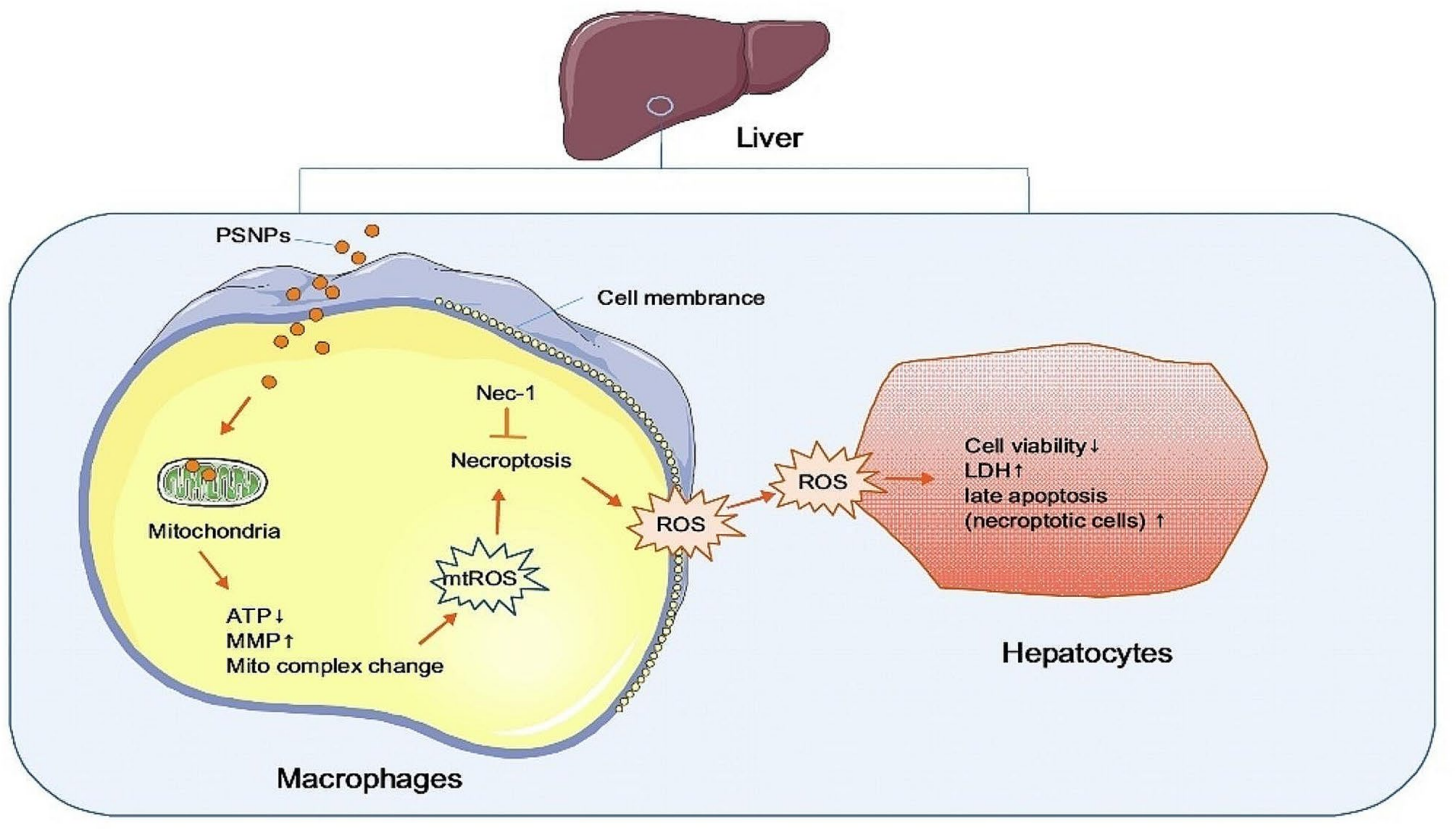
Schematic figure of this study

It is evident that exposure to environmentally degraded and synthetic PSNPs has become ubiquitous, activating multiple toxic pathways that promote cell death and organ injury in living organisms [22]. Therefore, a comprehensive assessment of the harmful consequences of PSNPs exposure and identification of the underlying mechanisms is essential. The key finding of this study underscores the hepatic toxic effect of acute exposure to 20 nm synthetic PSNPs, inducing redox-sensitive necroptosis in macrophages and upregulating the crosstalk between macrophages and hepatocytes (Fig. 10).

The intrinsic toxicity of NPs depends largely on their size and surface properties, which can dramatically affect their uptake and distribution in mammalian cells [23]. Existing studies have compared plastic particles ranging from tens to thousands of nanometers, demonstrating that smaller particles are more likely to be internalized by cells (e.g., epithelial cells and macrophages), accordingly triggering cytotoxicity to a greater extent [5, 24]. Consistent with previous findings, our study found that the viability of murine macrophages was significantly suppressed by 20 nm PSNPs but not affected by 100 or 1000 nm PS particles, indicating the preferential uptake and more severe toxicity of smaller PS (< 100 nm) than larger particles (≥ 100 nm) in monocytes or macrophages [18, 25, 26]. Surface chemistry also affects the toxic behavior of PS particles. For example, RAW 264.7 cells were shown to be tolerant to 60 nm plain PS while 60 nm amidinated PS dramatically disrupted cell viability after 16 or 24 h incubation [18, 26]. Furthermore, positively charged 20 nm PSNPs were more toxic than unmodified and negatively charged PSNPs when treated in murine splenocytes [18]. Our experiments yielded more severe results that treatment with 20 nm plain PS obviously triggered cytotoxicity by only 4 h treatment, highlighting the augmented toxicity towards macrophages after acute exposure to smaller PS particles. Of note, the toxic doses of PSNPs (50 µg/ml) in our study seem higher than the reported concentration of PSNPs (4.8 µg/mL) detected in the blood of human volunteers [27]. Our experiments yielded more severe results that treatment with 20 nm plain PS obviously triggered cytotoxicity by only 4 h treatment, highlighting the augmented toxicity towards macrophages after acute exposure to smaller PS particles. Of note, PS is regarded as derived from plastic PSLT materials while our study of another PSLT demonstrated different results of size-or surface area-dependent toxic effects [28]. This further suggest PSLT materials may augment their toxicity when becoming smaller enough to possess much increased total dosed surface area. However, the nature of materials also matters as PSNPs are far more toxic than TiO2 at the same size and dose. Therefore, our findings are meaningful by implicating that environmental or synthetic NP materials may become hazardous when the particles are small enough, irrespective of their different physicochemical properties [29].

The toxicity of nanomaterials is closely linked to their dosage. In our study, we employed an acute and high-dose exposure pattern of polystyrene nanoparticles (PSNPs) in both cellular (50 µg/ml) and animal (60 mg/kg) experiments to mimic the conditions of enhanced environmental exposure. These doses were also comparable to those used in previous published work [30–32]. Of note, recent studies considered a measurable concentration of 40 µg/ml NP in the aquatic environment. We thus suggest the use of 50 µg/ml PSNPs as an environment-related dose in cellular experiments [31]. To further establish their environmental or occupational relevance, we mathematically converted a typical 1 mm^3^ microplastics (MPs) into NPs using a previously established method [33]. This calculation yielded an estimated 2.4 × 10^14^ items/m^3^ of 20 nm PSNPs for each MP. Considering the estimated individual inhalation rate of MPs at 170 per day (equivalent to 4.08 × 10^16^ of 20 nm nanoparticles), we determined that the appropriate experimental exposure dose for mice was 5.8 × 10^13^ nanoparticles [34] due to the 704-fold difference in specific surface area between human lungs and experimental mice. Therefore, we used 60 mg/kg as the experimental exposure dose in vivo, which equates to 27 × 10^13^ total nanoparticles (volume × concentration × number of 1 mg nanoparticles), approximately 5 times the actual estimated exposure dose. Since the human burden of MPs or NPs could be significantly increased under certain conditions, research on acute and high doses of NP exposure is thus of great significance. It should be noted that the toxic doses of PSNPs (50 µg/ml) in our study were still higher than the reported concentration of PSNPs (4.8 µg/mL) detected in the blood of human volunteers [27]. However, our study focused on much smaller particles (20 nm) than their beads (700 nm), which can increase in amount by degrading from larger particles. In addition, circulatory NPs are likely to dramatically increase upon enhanced environmental exposure to plastic particles by activity intensity and behavioral types, such as seafood consumption, exposure by inhalation, dust/soil ingestion and occupational exposure to microplastic or nanoplastic dust with 100 times higher than ambient air quality standards [35, 36]. Furthermore, NPs may potentially accumulate in major organs such as the heart, lung, liver, and spleen, leading to elevated tissue concentrations that may exceed the threshold of inducing cytotoxicity. Indeed, it is reported that 30–99% of nanoparticles accumulate and sequester in the liver after administration into the body [33]. Moreover, a recent study by Yang et al. found that MP counts in cardiac tissue samples were at least 10 times higher than those in blood samples from the same subjects, raising concerns of potential organ accumulation [37].

Environmental chemicals and pollutants and synthetic nanomaterials can influence different routes of cell destiny, such as apoptosis, necroptosis, pyroptosis, ferroptosis, and autophagic cell death, depending largely on the exact cell context and types of stimuli [38–40]. However, data derived from viability experiments regarding cytotoxicity cannot distinguish the specific mode of cell death [39, 41]. In our study, we demonstrated that PSNPs triggered lytic cell death in murine macrophages through morphology observation and LDH release detection. Furthermore, we verified that PSNPs selectively induced necroptosis rather than other forms of lytic cell death in macrophages by conducting screening experiments with different cell death inhibitors and direct detection of necroptosis-specific pathways. To the best of our knowledge, our study was the first to reveal that 20 nm PSNPs independently and rapidly induce necroptosis in macrophages, although previous studies have implicated the induction of apoptosis [42, 43] or autophagic cell death [18] by PSNPs in macrophages or monocytes. The discrepancies are likely attributed to the relatively larger (40–60 nm) and modified (amidation) particles used in the reported studies. Furthermore, these studies did not actually exclude the existence of necroptosis by only detecting the common indicators rather than those that distinguish a specific form of cell death. Interestingly, we also observed that PSNPs-induced cytotoxicity was augmented when apoptosis was inhibited, highlighting a concomitant effect of necroptosis induction and the transition from apoptosis to necroptosis that augments the necroptotic cell death. Given the preferential uptake of nanoplastics by macrophages and the significance of macrophage necroptosis in inducing tissue injury, our findings are meaningful as they implicate a potential detrimental effect of necroptotic death induction after exposure to nanoplastics.

The molecular machinery mediated by tumor necrosis factor receptor 1 (TNFR1) remains one of the best-characterized controlling components through which nanoparticles induce necroptosis [40]. Our study has shown that PSNPs do not increase a significant TNF-α production. Additionally, it is worth noting that TNF-α typically induces necroptosis in macrophages at a final concentration of 30 ng/ml, which is considerably higher than the amount produced by PSNPs stimulation in our study (< 100 pg/ml) [44]. Thus, we suggested that TNF-α production may not depend directly on the master initiator of TNF or TNFR1 ligation to induce necroptosis. Accumulating evidence indicates that cellular exposure to nanoparticles generates ROS, which serves as another important inducer of necroptosis [45]. Our results have clearly demonstrated that PSNPs significantly disrupt the redox balance and upregulate ROS in macrophages, which essentially mediates the initiation of necroptosis. Although it is known that PS particles induce ROS generation in macrophages, the source of ROS has not been clearly clarified in these studies [18, 46]. In the current study, we have identified that mitochondrial ROS are mainly increased in PSNPs-exposed macrophages. Moreover, we have found that macrophage necroptosis is markedly inhibited when mtROS production is interrupted. It has become apparent that the generation of mtROS plays a critical role in the induction of necroptosis, including promoting RIP1 autophosphorylation [20], facilitating necrosome formation [45] and priming the switch from apoptosis to necroptosis [47]. However, there are no existing data that have either reported the upregulation of mtROS or demonstrated the involvement of mtROS in necroptosis induction upon PSNPs exposure in macrophages. In this regard, our results may suggest a potential new regulatory loop between PSNPs exposure, mtROS generation, and necroptosis induction.

It is known that nanoparticles are preferentially internalized by professional phagocytes, such as macrophages [48]. Previous data have apparently suggested that 20 nm PSNPs are taken up more rapidly and extensively than larger particles in macrophages [48, 49]. Our results have confirmed these findings by illustrating a time-dependent uptake of PSNPs, which was initiated as quickly as 10 min of incubation and became evident by 1 h post-treatment. Importantly, such rapid uptake of PSNPs was in accordance with the rapid inhibition of cell viability, potentially explaining their acute cytotoxicity in macrophages. Of note, the density of PSNPs is 1.05 g/cm^3^ according to literal data [50] and the physicochemical property data of PSNPs used in our study, which is equal to the densities of blood or other body fluids [51]. Thus, PSNPs can access and contact with macrophages rather than buoyant on the surface. This may further support the uptake of PSNPs in macrophages. The subcellular distribution of PSNPs in major organelles may critically decide the fate of particles and affect their cytotoxic effect [52, 53]. PSNPs typically accumulate in the lysosomes of macrophages and other parenchyma cells after cellular uptake, where they may disrupt lysosomal membranes and release lysosomal content that affects the integrity and function of other organelles [30]. Nonetheless, it is not clear whether PSNPs can subsequently invade other subcellular compartments before causing organelle damage and cell death. By using the fluorescence colocalization technique and TEM, we have demonstrated that 20 nm plain PSNPs are distributed in mitochondria rather than lysosomes after 4 h of treatment, and cause mitochondria with fractured and fuzzy cristae. This may also explain why PSNPs induce the generation of mtROS and thus promote necroptosis with a mitochondrial origin.

According to current literature reports, the possible mechanisms of their mitochondrial localization may be as follows. Firstly, PSNPs may enter mitochondria due to lysosomal destruction. It has been found that internalized PSNPs can be released by promoting lysosomes swelling and rupture in intestinal epithelial cells [2]. Wang et al. have also verified this process using a time-resolved method [53]. They have demonstrated that 50 nm PSNPs accumulate in lysosomes of 3–6 h post-exposure, leading to lysosomal swelling and rupture. This promotes PSNPs release into the cytoplasm and causes mitochondrial damage. Secondly, PSNPs may competitively bind with structural molecules of mitochondrial complex. Through molecular docking and molecular dynamics simulation, Huang et al. have found that compared with other types of nanoplastics, such as polypropylene (PP), polyethylene (PE), and polyvinyl chloride (PVC), the affinity score between PSNPs and the mitochondrial complex is the highest [54]. Competitive binding of PSNPs at the active site inhibits electron transfer, resulting in changes in the three-dimensional structure of the coenzyme Q10 binding domain, which directly affects mitochondrial oxidative phosphorylation (OXPHOS) and leads to reduced ATP synthesis. While these findings strongly support the hypothesis that PSNPs can enter mitochondria and inhibit mitochondrial complex, further experimental studies are needed to conclusively establish a direct cause-and-effect relationship.

Mitochondria play a pivotal role in cellular functions by oxidizing substrates and generating ATP, and mitochondrial dysfunction results in decreased oxygen consumption, impaired ATP synthesis, and increased electron leakage that generates mitochondrial reactive oxygen species (mtROS) [55]. In this study, we observed that increased mitochondrial accumulation led to severe mitochondrial dysfunction, including perturbed activation of complex I on the electron transport chain, membrane hyperpolarization, and abrupt ATP generation, which eventually favored the upregulation of mtROS to trigger necroptosis. Previous results have indicated that polystyrene nanoparticles (PSNPs) disrupt the integrity of lysosomes [26, 53], and it is reasonable to assume that PSNPs may be leaked from lysosomes and then enriched in mitochondria where they initiate necroptosis. However, other direct or indirect routes or mechanisms of PSNPs mitochondrial accumulation cannot be excluded. For example, we also observed the distribution of PSNPs in the endoplasmic reticulum, which warrants future research to determine whether PSNPs can also conquer mitochondria or otherwise trigger cell death via ER-dependent mechanisms. Nevertheless, our study may still suggest a direct mitochondrial contact mechanism by which PSNPs induce mitochondrial damage and mtROS generation that eventually promote necroptosis.

The liver serves as the primary metabolic site for the clearance of PSNPs, and frequent interactions with PS particles can lead to many detrimental effects, including excessive inflammation, cell death, and aberrant lipid metabolism [56]. In our study, the doses of PSNPs were set at 60 mg/kg. bw (equivalent to 3.6 g for an adult weighing 60 kg), aiming to assess the acute toxicity to humans with high NP exposure [57, 58]. We found that acute exposure to 20 nm PSNPs significantly aggravated liver injury in mice, along with the disruption of redox balance in hepatic tissues. Similar findings have been reported by Ding et al. [30], Fan et al. [59], Deng et al. [60] and Choi et al. [32], but future studies are needed to comprehensively characterize the toxicity of 20 nm PSNPs at the molecular level.

Importantly, the hepatotoxic effect and oxidative stress due to PSNPs administration were abolished by concomitant treatment with Nec-1, a selective necroptosis inhibitor. Our results thus confirm the hepatotoxic effect of PSNPs while highlighting a new mechanism involving the induction of hepatic necroptosis. Furthermore, liver injury can be induced by either direct hepatotoxicity or crosstalk between macrophages and hepatocytes [13]. However, it remains to be clarified how PSNPs promote liver injury. We discovered that 20 nm plain PSNPs were highly toxic in macrophages but did not affect the viability of either murine (AML-12) or human (HuH7) hepatocytes. An early study similarly revealed that NH2-labeled PS nanospheres demonstrated toxicity in RAW 264.7 cells rather than endothelial, hepatoma, or pheochromocytoma cells [26]. Although PSNPs did not induce cytotoxicity in hepatocytes directly, our results indicated that PSNPs may upregulate ROS generation and trigger cell death in AML-12 cells when cocultured with RAW 264.7 cells. The requirement of macrophage-dependent intercellular crosstalk in mediating hepatocyte injury in vitro was further confirmed by macrophage depletion approaches in vivo, which indicates a previously unknown mechanism by which hepatic macrophages and their necroptosis essentially mediate liver injury due to PSNPs administration. In a recent study, we similarly observed that gold nanoparticles aggravated liver injury by amplifying ROS-mediated macrophage-hepatocyte crosstalk [13]. Our findings may thus implicate an interactive mechanism between macrophages and hepatocytes that may universally exist to drive the hepatoxic behaviors of nanoparticles.

Some limitations exist in this study and should be noted. Firstly, we used acute and high-dose of PSNPs to study their toxicity. Although we have explained the rationale of this exposing pattern, humans are more often exposed to low-dose MNPs repeatedly. Therefore, a low-dose and long-term exposure should be also necessary to better evaluate the harmful effects of PSNPs in a more realistic manner. Secondly, published work has always used synthetic NP beads without considering their relevance with MNPs in environmental or occupational settings [61]. The inert nature of environmental and synthetic NPs may be the same. However, synthetic NPs are uniform while environmental MNPs are much more complex in sizes, materials, and durations [62]. As it is particularly difficult to separate small-sized NPs from environmental samples and quantify their amounts, we have to use synthetic NPs currently. Another environmental approach is to use aged particles. Although we have found that fresh PSNPs are structurally identical with aged PSNPs, the latter may exhibit stronger toxicity (data not shown). Therefore, it is noteworthy that environmental MNPs are far more complex in composition and toxic behaviors than synthetic particles. Thirdly, we only used fluorescence methods to trace the intracellular localization of PSNPs in macrophages. Other label-free methods are also encouraged to be employed which may indicate its distribution more directly.

## Conclusion

In summary, our investigation unveils the detrimental impact of acute exposure to 20 nm plain PSNPs on liver health by inducing necroptosis in macrophages and intensifying the interplay between macrophages and hepatocytes. Our findings reveal that PSNPs are swiftly internalized by macrophages and accumulate in mitochondria, causing mitochondrial harm and upregulating mtROS generation. This process ultimately triggers macrophage necroptosis, leading to intercellular crosstalk and consequent hepatocyte damage. Our study sheds light on the profound immunotoxicity of environmental PSNPs exposure and their associated hepatotoxic effects through intercellular crosstalk in the hepatic microenvironment.

## Materials and methods

### Characterizations of PSMPs/PSNPs

Polystyrene nanoparticles (PSNPs) with diameters of 20 and 100 nm, FITC-labeled PSNPs (fPSNPs, 20 nm), Nile Red-labeled PSNPs (20 nm) and polystyrene microplastics (PS-MPs, 1 μm) were obtained from XFNANO Materials Technology Co., Ltd. (Nanjing, China). The operation steps refer to the literature, briefly, the styrene is purified, and the styrene monomer emulsion is polymerized into polystyrene nanoplastics [63]. High-resolution images for the characterization of PSNPs/PSMPs were captured using a transmission electron microscope (TEM, JEOL 1011, Japan). The particle size distribution and zeta potential of PSNPs/PSMPs dispersed in deionized (DI) water and DMEM (Gibco, USA) were determined using a Zetasizer Nano ZS (Malvern Instruments, Worcestershire, UK).

### Chemicals

Chemicals The following inhibitors and reagents were purchased: Necrostatin-1 (Nec-1, 50 µM), GSK’843 (1 µM), necrosulfonamide (NSA, 1 µM), MCC950 (1 µM), wortmannin (Wort, 100 nM), 3-methyladenine (3-MA, 5 mM), Z-VAD-FMK (40 µM), Ferrostatin-1 (Fer-1, 5 µM), deferoxamine (DFO, 5 µM), mito-TEMPO (10 µM), and NAC (400 µM) from MCE (NJ, USA) or Sigma Aldrich (MO, USA). TiO2 (20 nm, 100 nm and 1 μm) were obtained from XFNANO Materials Technology Co., Ltd. (Nanjing, China). The 2 g TiO2 and 1 g sodium hexametaphosphate (Aladdin ®, Shanghai, China) were added to double distilled water, mixed well and diluted to 100 ml, ultrasonically oscillated at 4 °C for 15 min to prepare 20 µg/µl TiO2 dispersion. The TiO2 dispersion was sterilized by high pressure steam at 121 °C for 30 min.

### Animals

Wild-type BALB/c mice (male, 6–8 weeks old) were acquired from HFK Bioscience Co., Ltd. (Beijing, China). Mice were housed in a specific pathogen-free (SPF) environment, maintained at 22 °C with 50–60% humidity, and provided with a standard diet. All animal experiments were conducted in accordance with national and institutional guidelines for animal care and use, and were approved by the Institutional Animal Ethics Committee of the Third Military Medical University.

### Cell culture

Bone marrow-derived macrophages (BMDMs) and peritoneal macrophages were isolated from the hind femora, tibias, or peritoneum of BALB/c mice and cultured as previously described [64, 65]. The murine macrophage cell line RAW 264.7, J774A.1, and the human hepatocarcinoma cell line (Huh7) were cultured in DMEM supplemented with 10% fetal bovine serum (FBS, Hyclone, UT, USA). The murine normal hepatocyte cell line alpha mouse liver 12 (AML-12) was cultured in DMEM/nutrient mixture F-12 (DMEM-12, Gibco, USA) containing 1% ITS Liquid Media Supplement (Sigma), 10% FBS, and 40 ng/ml dexamethasone (Sigma). THP-1 cells were cultured in Roswell Park Memorial Institute (RPMI) 1640 medium (Gibco, USA) supplemented with 10% FBS. AML-12 and J774A.1 cells were obtained from Procell Life Science & Technology Co., Ltd. (Wuhan, China), while RAW 264.7 and THP-1 cell lines were purchased from the American Type Culture Collection (USA). Huh7 cells were kindly provided by Dr. Haojun Xiong, Department of Hepatobiliary Surgery, Southwest Hospital, Army Military Medical University, China. All cell lines were maintained at a concentration of 1 × 10^5^ to 1 × 10^6^ cells/ml in a humidified atmosphere of 5% CO2 at 37 °C.

### Coculture experiments

RAW 264.7 cells and AML-12 cell monolayers were separately seeded into the upper and lower compartments of a Transwell chamber (Corning, China) with a pore diameter of 3 μm before treatment with PSNPs. During the experiment, AML-12 cells were treated with PSNPs alone, or with supernatants from RAW 264.7 cells treated with PSNPs for 4 h, which were obtained via cocultivation.

### Cell internalization detection

The ability of cells to internalize fPSNPs was assessed by flow cytometry (ACEA NovoCyte, USA) and Zeiss 780 laser scanning confocal microscopy (LSCM) (Zeiss, Germany). Cells were treated with 50 µg/mL of fPSNPs for 0–4 h. After treatment, the cells were collected, centrifuged, and resuspended in Hank’s balanced salt solution (HBSS, Sigma Aldrich). Intracellular fluorescence was measured with excitation/emission spectra at 488/520 nm.

### Detection of the subcellular distribution of PSNPs

To investigate the subcellular distribution of PSNPs, RAW 264.7 cells (1 × 10^5^ cells/mL) or J774A.1 cells (1 × 10^5^ cells/mL) were incubated with 50 µg/mL fPSNPs or Neil-red PSNPs for 4 h, followed by co-incubation with fluorescent dyes, such as MitoBright LT Red (Dojindo Molecular Technologies, Japan), LysoTracker Red, ER-Tracker Red, and 1,1’-dioctadecyl-3,3,3’,3’-tetramethylindocarbocyanine perchlorate (Dil) (Beyotime, China) for 20 min. After washing with PBS, the cells were then stained with 2-(4-amidinophenyl)-6-indolecarbamidine dihydrochloride (DAPI) solution (Beyotime) at room temperature for 10 min and subjected to confocal microscopy to measure the fluorescence intensities. Moreover, the localization and mitochondria ultrastructure change of PS in RAW264.7 cells and J774A.1 cells were evaluated by TEM (JEOL 1011, Japan).

### Assessment of cell viability using CCK-8 assay

Cell viability was evaluated using the cell counting kit (CCK-8) assay (Dojindo) as previously described [66]. RAW 264.7 cells were seeded on 96-well plates at a density of 1 × 10^5^ cells/well and exposed to a range of concentrations from 0 µg/mL to 100 µg/mL of PSNPs for 0–4 h to perform the toxicity assessment.

### LDH release assay for cell death detection

The LDH released into cell culture supernatants was detected using an LDH assay kit (Beyotime). Independently cultured AML-12 cells, RAW 264.7 cells, or a coculture of AML-12 cells with RAW 264.7 cells in a transwell system were treated with PSNPs for 4 h and then processed according to the manufacturer’s instructions. The absorbance at 490 nm was detected with a Varioskan flash multimode reader (ThermoFisher).

### Quantification of ROS generation using flow cytometry

The intracellular generation of total ROS or the level of mtROS were measured by flow cytometry (ACEA NovoCyte, USA) using DCFH-DA (Sigma Aldrich) or MitoSOX Red staining (Molecular Probes, OR, USA) according to the manufacturers’ instructions.

### Annexin V-FITC/PI staining analysis for apoptosis and necrosis detection

The ratios of apoptotic and necrotic cells were quantified using the Annexin V-FITC/PI flow cytometry double staining method, which enables the clear discrimination of viable cells (FITC-/PI-), early apoptotic cells with intact cell membranes (FITC+/PI-), primary necrotic cells (FITC-/PI+), and late apoptosis/necroptosis cells (FITC+/PI+) [67, 68]. RAW 264.7 cells were treated with PSNPs at the designated concentrations for 4 h. Then, the cells were harvested, washed with PBS, stained with an Annexin V-FITC/PI apoptosis detection kit (BD Biosciences, CA, USA), and measured via flow cytometry (ACEA NovoCyte, USA).

### Western blot analysis for protein expression analysis

Homogenized animal tissues and treated cells were lysed with RIPA lysis buffer (Roche, Basel, Switzerland) containing protease inhibitors and phosphatase inhibitors. BCA protein assay kit (Beyotime) was used to measure protein concentrations. Proteins were separated by SDS-PAGE and transferred to polyvinylidene fluoride (PVDF) membranes, which were blocked for 1 h and then blotted with specific primary antibodies against actin, GAPDH, RIP1, RIP3, MLKL, phosphor (P)-RIP1, P-RIP3, and P-MLKL (1:1000 dilution; Cell Signaling Technology, MA, USA) overnight at 4℃. The membranes were washed with TBS-T several times and further incubated with the secondary antibodies (1:1000 dilution) at 37℃ for 1 h. Finally, chemiluminescence images were developed with a SuperSignal Sensitivity Substrate kit (Thermo Fisher Scientific, MA, USA) and detected using a ChemiDoc XRS + imaging system (Bio-Rad, CA, USA).

### ELISA for TNF-α detection

The concentration of TNF-α was measured with ELISA kits according to the manufacturer’s instructions (Invitrogen, CA, USA).

### Determination of oxidative stress levels

The enzymatic activities of GSH, SOD, -SH and MDA in the cells and tissues were quantified using commercial biochemical kits (Jiancheng Bioengineering Institute, Nanjing, China) according to the manufacturer’s instructions. Tissue enzyme activity was normalized to the protein content.

### Mitochondrial function detection

A mitochondrial respiratory chain complex I-V activity assay kit (Solarbio, Beijing, China) was used to detect the inhibition of mitochondrial respiratory electron transport chain activity in cells. The mitochondrial membrane potential (MMP) was examined using TMRM reagent (Invitrogen). ATP levels were detected using a luciferin and luciferase bioluminescence assay kit (Beyotime). The intensity of the chemiluminescent signal was measured with a Varioskan flash multimode reader.

### In vivo experiments on mice

BALB/c mice were intraperitoneally injected with 20 nm PSNPs (60 mg/kg) or normal saline (NS) for 24 h (*n* = 6 per group). The volume of one MPs is estimated to be 1mm^3^ [33]. According to the formula $\frac{(10^6nm)^3}{\frac{4}{3}\pi {r}^{3}}$ conversion, 1 MPs is deduced to be about 2.4 × 10^14^ items/m^3^ of 20 nm PSNPs [69]. Due to the average inhalation of 170 MPs per person per day (equivalent to 4.08 × 10^16^ 20 nm nanoparticles), the difference between the specific surface area of human lung (62.7 m^2^) and the specific surface area of experimental mice (body weight of about 20 g) (0.089 m^2^) is 704 times, the appropriate experimental exposure of mice is 5.8 × 10^13^ items nanoparticles [34]. However, in addition to inhaling MPs, normal adults are estimated to ingest an additional 142 MPs per person per day. In studies related to human consumption of MPs, seafood accounts for a large proportion. Considering that the annual consumption of seafood varies greatly among many urbanized populations, for example, the actual intake of American men aged 19–30 years is 125 g/week, while Japan’s seafood consumption is the highest, estimated at 104.2 g/day, with a difference of 5.8 times [70]. A study from New York University found that the MPs content in infant feces was 20 times higher than that in adults because infants tended to crawl on the floor and chew plastic toys to increase their contact with plastic. In addition, people are also exposed to higher concentrations of MPs in plastic-related workplaces, such as PVC production workshops, textile factory workshops, etc. [71]. Therefore, in vivo experimental exposure dose 60 mg/kg (27 × 10^13^ items), is about 5 times the actual estimated exposure dose, which is also possible in real life.

In other in vivo experiments, BALB/c mice were preinjected with clodronate liposomes (CLLs) (Liposoma, Amsterdam, The Netherlands) or control liposomes for 3 consecutive days and then challenged with PSNPs (60 mg/kg) for 24 h according to published papers (*n* = 3 per group) [72]. Blood samples were collected by cardiac puncture and the liver was eviscerated and sampled for histological examination.

### Histological examination of liver tissue sections

The liver tissue was embedded in 4% paraformaldehyde to prepare 5-µm paraffin sections and 10-µm frozen sections for hematoxylin-eosin staining (H&E) and oil red O staining (ORO) (Servicebio, Hubei, China), respectively. The histological morphology of liver sections was observed using a Nikon Eclipse E100 upright microscope (Nikon, Japan).

### Biochemical measurement

A biochemical blood analyzer (Labospect 008 AS, Japan) was used for blood biochemical analysis, including alanine aminotransferase (ALT), aspartate aminotransferase (AST). Lactic dehydrogenase (LDH) assay kits (Maccura Biotechnology Co., Ltd, Sichuan, China) were used for serum LDH detection.

### Immunofluorescence analysis

Frozen sections of liver tissue were prepared immediately after sampling and then incubated with F4/80 (1:300, CST, USA) antibodies at 4℃ overnight. The sections were incubated with Cy3-conjugated secondary antibodies (1:300, Beyotime) and stained with DAPI (Beyotime). Finally, a ZEISS 780 laser confocal microscope was used to capture fluorescence images and analyze macrophage clearance by immunofluorescence staining.

### Statistical analysis

All data are presented as the mean ± standard error of the mean (SEM). Statistical analysis was performed using unpaired Student’s t-test or one-way ANOVA followed by Dunnett post hoc test in GraphPad Prism 8. Statistical significance was defined as *P ≤ 0.05, **P ≤ 0.01.

### Electronic supplementary material

Below is the link to the electronic supplementary material.

Supplementary Material 1

Supplementary Material 2

## Data Availability

All data generated or analyzed during this study are available from the corresponding author.

## References

[1] Banerjee A, Shelver WL. Micro- and nanoplastic induced cellular toxicity in mammals: a review. Sci Total Environ Sci Total Environ; 2021;755. 10.1016/j.scitotenv.2020.142518 33065507

[2] Xu D, Ma Y, Peng C, Gan Y, Wang Y, Chen Z (2023). Differently surface-labeled polystyrene nanoplastics at an environmentally relevant concentration induced Crohn’s ileitis-like features via triggering intestinal epithelial cell necroptosis. Environ Int.

[3] Yang S, Cheng Y, Chen Z, Liu T, Yin L, Pu Y (2021). In vitro evaluation of nanoplastics using human lung epithelial cells, microarray analysis and co-culture model. Ecotoxicol Environ Saf.

[4] Dick Vethaak A, Legler J (2021). Microplastics and human health. Science.

[5] Schröter L, Ventura N (2022). Nanoplastic toxicity: insights and challenges from experimental Model systems. Small.

[6] Wang S, Zhang R, Wang D (2021). Induction of protective response to polystyrene nanoparticles associated with methylation regulation in Caenorhabditis elegans. Chemosphere.

[7] Sun W, Jin C, Bai Y, Ma R, Deng Y, Gao Y (2022). Blood uptake and urine excretion of nano- and micro-plastics after a single exposure. Sci Total Environ.

[8] Deng J, Ibrahim MS, Tan LY, Yeo XY, Lee YA, Park SJ (2022). Microplastics released from food containers can suppress lysosomal activity in mouse macrophages. J Hazard Mater.

[9] Florance I, Chandrasekaran N, Gopinath PM, Mukherjee A (2022). Exposure to polystyrene nanoplastics impairs lipid metabolism in human and murine macrophages in vitro. Ecotoxicol Environ Saf Ecotoxicol Environ Saf.

[10] Busch M, Bredeck G, Waag F, Rahimi K, Ramachandran H, Bessel T (2022). Assessing the NLRP3 inflammasome activating potential of a large panel of Micro- and nanoplastics in THP-1 cells. Biomolecules.

[11] Zhang YN, Poon W, Tavares AJ, McGilvray ID, Chan WCW (2016). Nanoparticle-liver interactions: Cellular uptake and hepatobiliary elimination. J Control Release.

[12] Chang X, Xue Y, Li J, Zou L, Tang M (2020). Potential health impact of environmental micro- and nanoplastics pollution. J Appl Toxicol.

[13] Yang Y, Fan S, Chen Q, Lu Y, Zhu Y, Chen X (2022). Acute exposure to gold nanoparticles aggravates lipopolysaccharide-induced liver injury by amplifying apoptosis via ROS-mediated macrophage-hepatocyte crosstalk. J Nanobiotechnol.

[14] Auta HS, Emenike CU, Fauziah SH (2017). Distribution and importance of microplastics in the marine environment: a review of the sources, fate, effects, and potential solutions. Environ Int.

[15] Jing J, Zhang L, Han L, Wang J, Zhang W, Liu Z (2022). Polystyrene micro-/nanoplastics induced hematopoietic damages via the crosstalk of gut microbiota, metabolites, and cytokines. Environ Int.

[16] Teng M, Zhao X, Wu F, Wang C, Wang C, White JC (2022). Charge-specific adverse effects of polystyrene nanoplastics on zebrafish (Danio rerio) development and behavior. Environ Int.

[17] Nguyen B, Claveau-Mallet D, Hernandez LM, Xu EG, Farner JM, Tufenkji N (2019). Separation and analysis of Microplastics and nanoplastics in Complex Environmental samples. Acc Chem Res.

[18] Chiu HW, Xia T, Lee YH, Chen CW, Tsai JC, Wang YJ (2015). Cationic polystyrene nanospheres induce autophagic cell death through the induction of endoplasmic reticulum stress. Nanoscale.

[19] Li Y, Xu M, Zhang Z, Halimu G, Li Y, Li Y (2022). In vitro study on the toxicity of nanoplastics with different charges to murine splenic lymphocytes. J Hazard Mater.

[20] Zhang Y, Su SS, Zhao S, Yang Z, Zhong CQ, Chen X (2017). RIP1 autophosphorylation is promoted by mitochondrial ROS and is essential for RIP3 recruitment into necrosome. Nat Commun.

[21] Wu Y, Vadim Zinchuk 1 (2013). Bridging the gap between qualitative and quantitative colocalization results in fluorescence microscopy studies. Sci Rep.

[22] Fournier SB, D’Errico JN, Adler DS, Kollontzi S, Goedken MJ, Fabris L (2020). Nanopolystyrene translocation and fetal deposition after acute lung exposure during late-stage pregnancy. Part Fibre Toxicol.

[23] He Y, Li J, Chen J, Miao X, Li G, He Q (2020). Cytotoxic effects of polystyrene nanoplastics with different surface functionalization on human HepG2 cells. Sci Total Environ.

[24] Liu H, Tian L, Wang S, Wang D (2021). Size-dependent transgenerational toxicity induced by nanoplastics in nematode Caenorhabditis elegans. Sci Total Environ.

[25] Jeon S, Clavadetscher J, Lee DK, Chankeshwara SV, Bradley M, Cho WS (2018). Surface Charge-Dependent Cellular Uptake of Polystyrene nanoparticles. Nanomater (Basel Switzerland).

[26] Xia T, Kovochich M, Liong M, Zink JI, Nel AE (2008). Cationic polystyrene nanosphere toxicity depends on cell-specific endocytic and mitochondrial injury pathways. ACS Nano.

[27] Leslie HA, van Velzen MJM, Brandsma SH, Vethaak AD, Garcia-Vallejo JJ, Lamoree MH (2022). Discovery and quantification of plastic particle pollution in human blood. Environ Int.

[28] Cosnier F, Seidel C, Valentino S, Schmid O, Bau S, Vogel U (2021). Retained particle surface area dose drives inflammation in rat lungs following acute, subacute, and subchronic inhalation of nanomaterials. Part Fibre Toxicol.

[29] Wang F, Yu L, Monopoli MP, Sandin P, Mahon E, Salvati A (2013). The biomolecular corona is retained during nanoparticle uptake and protects the cells from the damage induced by cationic nanoparticles until degraded in the lysosomes. Nanomedicine.

[30] Ding Y, Zhang R, Li B, Du Y, Li J, Tong X (2021). Tissue distribution of polystyrene nanoplastics in mice and their entry, transport, and cytotoxicity to GES-1 cells. Environ Pollut.

[31] Lin S, Zhang H, Wang C, Su XL, Song Y, Wu P (2022). Metabolomics Reveal Nanoplastic-Induced mitochondrial damage in Human Liver and Lung cells. Environ Sci Technol.

[32] Choi YJ, Park JW, Lim Y, Seo S, Hwang DY (2021). In vivo impact assessment of orally administered polystyrene nanoplastics: biodistribution, toxicity, and inflammatory response in mice. Nanotoxicology.

[33] Fournier SB, D’Errico JN, Adler DS, Kollontzi S, Goedken MJ, Fabris L (2020). Nanopolystyrene translocation and fetal deposition after acute lung exposure during late-stage pregnancy. Part Fibre Toxicol.

[34] Wu Y, Yao Y, Bai H, Shimizu K, Li R, Zhang C (2023). Investigation of pulmonary toxicity evaluation on mice exposed to polystyrene nanoplastics: the potential protective role of the antioxidant N-acetylcysteine. Sci Total Environ.

[35] Liu K, Li Q, Andrady AL, Wang X, He Y, Li D (2023). Underestimated activity-based microplastic intake under scenario-specific exposures. Environ Sci Ecotechnol.

[36] Zarus GM, Muianga C, Hunter CM, Pappas RS (2021). A review of data for quantifying human exposures to micro and nanoplastics and potential health risks. Sci Total Environ.

[37] Yang Y, Xie E, Du Z, Peng Z, Han Z, Li L (2023). Detection of Various Microplastics in Patients Undergoing Cardiac Surgery. Environ Sci Technol.

[38] Franco R, Panayiotidis MI (2010). Cell death or survival: the double-edged sword of environmental and occupational toxicity. Chem Biol Interact.

[39] Mohammadinejad R, Moosavi MA, Tavakol S, Vardar DÖ, Hosseini A, Rahmati M (2019). Necrotic, apoptotic and autophagic cell fates triggered by nanoparticles. Autophagy.

[40] Sepand MR, Aliomrani M, Hasani-Nourian Y, Khalhori MR, Farzaei MH, Sanadgol N (2020). Mechanisms and pathogenesis underlying environmental chemical-induced necroptosis. Environ Sci Pollut Res Int.

[41] Qiao R, Mortimer M, Richter J, Rani-Borges B, Yu Z, Heinlaan M (2022). Hazard of polystyrene micro-and nanospheres to selected aquatic and terrestrial organisms. Sci Total Environ.

[42] Hu Q, Wang H, He C, Jin Y, Fu Z (2021). Polystyrene nanoparticles trigger the activation of p38 MAPK and apoptosis via inducing oxidative stress in zebrafish and macrophage cells. Environ Pollut.

[43] Ilić K, Kalčec N, Krce L, Aviani I, Turčić P, Pavičić I (2022). Toxicity of nanomixtures to human macrophages: joint action of silver and polystyrene nanoparticles. Chem Biol Interact.

[44] Shi F, li, Yuan L, sha, Wong T, sui, Li Q, Li Y ping, Xu R et al. Dimethyl fumarate inhibits necroptosis and alleviates systemic inflammatory response syndrome by blocking the RIPK1-RIPK3-MLKL axis. Pharmacol Res. 2023:189:106697. 10.1016/j.phrs.2023.106697 36796462

[45] Weindel CG, Martinez EL, Zhao X, Mabry CJ, Bell SL, Vail KJ (2022). Mitochondrial ROS promotes susceptibility to infection via gasdermin D-mediated necroptosis. Cell.

[46] Xia T, Kovochich M, Brant J, Hotze M, Sempf J, Oberley T (2006). Comparison of the abilities of ambient and manufactured nanoparticles to induce cellular toxicity according to an oxidative stress paradigm. Nano Lett.

[47] Deragon MA, McCaig WD, Patel PS, Haluska RJ, Hodges AL, Sosunov SA (2020). Mitochondrial ROS prime the hyperglycemic shift from apoptosis to necroptosis. Cell Death Discov.

[48] Dos Santos T, Varela J, Lynch I, Salvati A, Dawson KA (2011). Quantitative assessment of the comparative nanoparticle-uptake efficiency of a range of cell lines. Small.

[49] Clift MJD, Rothen-Rutishauser B, Brown DM, Duffin R, Donaldson K, Proudfoot L (2008). The impact of different nanoparticle surface chemistry and size on uptake and toxicity in a murine macrophage cell line. Toxicol Appl Pharmacol.

[50] Leiser R, Wu GM, Neu TR, Wendt-Potthoff K. Biofouling, metal sorption and aggregation are related to sinking of microplastics in a stratified reservoir. Water Res. 2020; 176. 10.1016/j.watres.2020.115748 32247995

[51] Vitello DJ, Ripper RM, Fettiplace MR, Weinberg GL, Vitello JM. Blood Density Is Nearly Equal to Water Density: A Validation Study of the Gravimetric Method of Measuring Intraoperative Blood Loss. J Vet Med. 2015; 2015:1–4. 10.1155/2015/152730 PMC4590883 26464949

[52] Liu L, Xu K, Zhang B, Ye Y, Zhang Q, Jiang W. Cellular internalization and release of polystyrene microplastics and nanoplastics. Sci Total Environ. 2021;779. 10.1016/j.scitotenv.2021.146523 34030247

[53] Wang F, Bexiga MG, Anguissola S, Boya P, Simpson JC, Salvati A (2013). Time resolved study of cell death mechanisms induced by amine-modified polystyrene nanoparticles. Nanoscale.

[54] Huang Y, Liang B, Li Z, Zhong Y, Wang B, Zhang B (2023). Polystyrene nanoplastic exposure induces excessive mitophagy by activating AMPK/ULK1 pathway in differentiated SH-SY5Y cells and dopaminergic neurons in vivo. Part Fibre Toxicol.

[55] Kumar A, Davuluri G, Welch N, Kim A, Gangadhariah M, Allawy A (2019). Oxidative stress mediates ethanol-induced skeletal muscle mitochondrial dysfunction and dysregulated protein synthesis and autophagy. Free Radic Biol Med.

[56] Bartucci R, Åberg C, Melgert BN, Boersma YL, Olinga P, Salvati A (2020). Time-resolved quantification of nanoparticle uptake, distribution, and Impact in Precision-Cut liver slices. Small.

[57] Shan S, Zhang Y, Zhao H, Zeng T, Zhao X (2022). Polystyrene nanoplastics penetrate across the blood-brain barrier and induce activation of microglia in the brain of mice. Chemosphere.

[58] Senathirajah K, Attwood S, Bhagwat G, Carbery M, Wilson S, Palanisami T (2021). Estimation of the mass of microplastics ingested– a pivotal first step towards human health risk assessment. J Hazard Mater.

[59] Fan X, Wei X, Hu H, Zhang B, Yang D, Du H (2022). Effects of oral administration of polystyrene nanoplastics on plasma glucose metabolism in mice. Chemosphere.

[60] Deng Y, Zhang Y, Lemos B, Ren H (2017). Tissue accumulation of microplastics in mice and biomarker responses suggest widespread health risks of exposure. Sci Rep.

[61] Martin LMA, Gan N, Wang E, Merrill M, Xu W (2022). Materials, surfaces, and interfacial phenomena in nanoplastics toxicology research. Environ Pollut.

[62] Wang B, Liang B, Huang Y, Li Z, Zhang B, Du J (2023). Long-Chain Acyl Carnitines Aggravate Polystyrene nanoplastics-Induced atherosclerosis by Upregulating MARCO. Adv Sci (Weinh).

[63] Xiong Y, Zhao J, Li L, Wang Y, Dai X, Yu F (2020). Interfacial interaction between micro/nanoplastics and typical PPCPs and nanoplastics removal via electrosorption from an aqueous solution. Water Res.

[64] Liu X, Wang N, Fan S, Zheng X, Yang Y, Zhu Y (2016). The citrus flavonoid naringenin confers protection in a murine endotoxaemia model through AMPK-ATF3-dependent negative regulation of the TLR4 signalling pathway. Sci Rep.

[65] Zeng CY, Li CG, Shu JX, Xu LH, Ouyang DY, Mai FY (2019). ATP induces caspase-3/gasdermin E-mediated pyroptosis in NLRP3 pathway-blocked murine macrophages. Apoptosis.

[66] Im GB, Kim YG, Jo IS, Yoo TY, Kim SW, Park HS (2022). Effect of polystyrene nanoplastics and their degraded forms on stem cell fate. J Hazard Mater.

[67] Lu B, Gong X, Wang ZQ, Ding Y, Wang C, Luo TF (2017). Shikonin induces glioma cell necroptosis in vitro by ROS overproduction and promoting RIP1/RIP3 necrosome formation. Acta Pharmacol Sin.

[68] Park J, Kim H, Do, Lee SH, Kwak CH, Chang YC, Lee YC (2019). Ascochlorin induces caspase-independent necroptosis in LPS-stimulated RAW 264.7 macrophages. J Ethnopharmacol.

[69] Wu Y, Wang J, Zhao T, Sun M, Xu M, Che S (2024). Polystyrene nanoplastics lead to ferroptosis in the lungs. J Adv Res.

[70] Cox KD, Covernton GA, Davies HL, Dower JF, Juanes F, Dudas SE (2019). Human consumption of Microplastics. Environ Sci Technol.

[71] Yin J, Ju Y, Qian H, Wang J, Miao X, Zhu Y (2022). Nanoplastics and Microplastics May be damaging our livers. Toxics.

[72] Chen J, Zhong MC, Guo H, Davidson D, Mishel S, Lu Y (2017). SLAMF7 is critical for phagocytosis of haematopoietic tumour cells via Mac-1 integrin. Nature.

