# Supplementary material for: Acute exposure to polystyrene nanoparticles promotes liver injury by inducing mitochondrial ROS-dependent necroptosis and augmenting macrophage-hepatocyte crosstalk
Source: Part Fibre Toxicol. 2024 Apr 12;21:20. doi: 10.1186/s12989-024-00578-6 (PMC11010371; doi:10.1186/s12989-024-00578-6)
Supplement: Supplementary file 1 — Supplementary Material 1 [file 12989_2024_578_MOESM1_ESM.pdf]

Western Blot Overview

Acute exposure to polystyrene nanoparticles promotes liver injury by inducing mitochondrial ROS-dependent necroptosis and augmenting macrophage-hepatocyte crosstalk

Junjie Fan, Li Liu, Yongling Lu, Qian Chen, Shijun Fan, Yongjun Yang, Yupeng Long, Xin Liu

Notes: The gels were physically cut to allow incubation with different antibodies targeting proteins with distinct molecular weights. These fragments are shown in the subsequent slides. Samples are loaded in pairs, with molecular weight markers in the first or last lanes. Only samples that were used in the representative blots are marked with red boxes.

Figure 3D. PSNP-treated RAW264.7 cell at the different time.

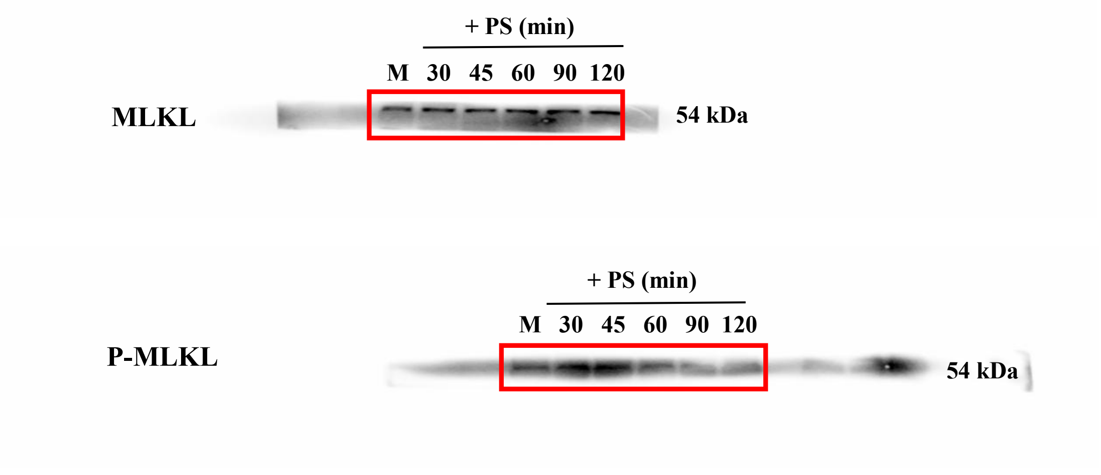

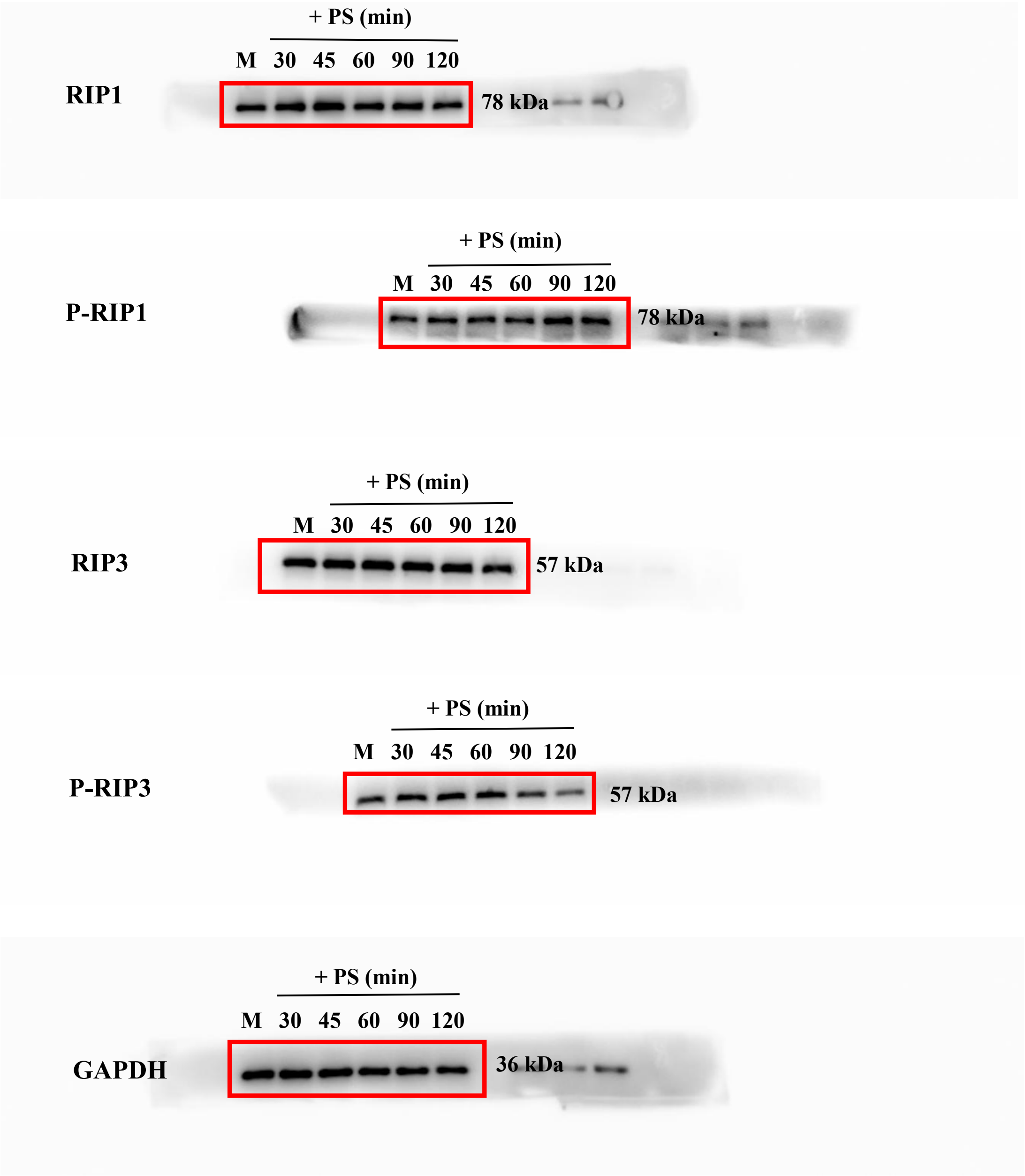

Figure 3D. PSNP-treated RAW 264.7 cells with Nec-1.

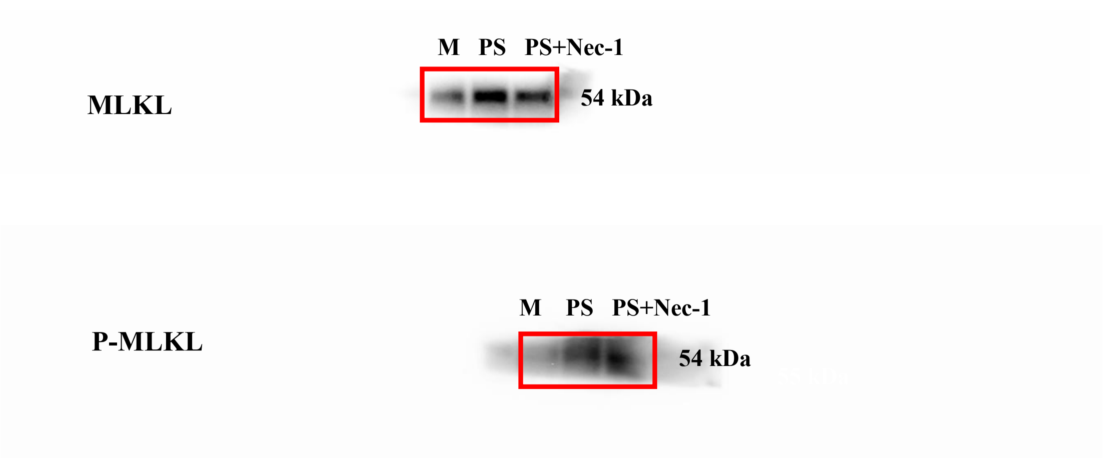

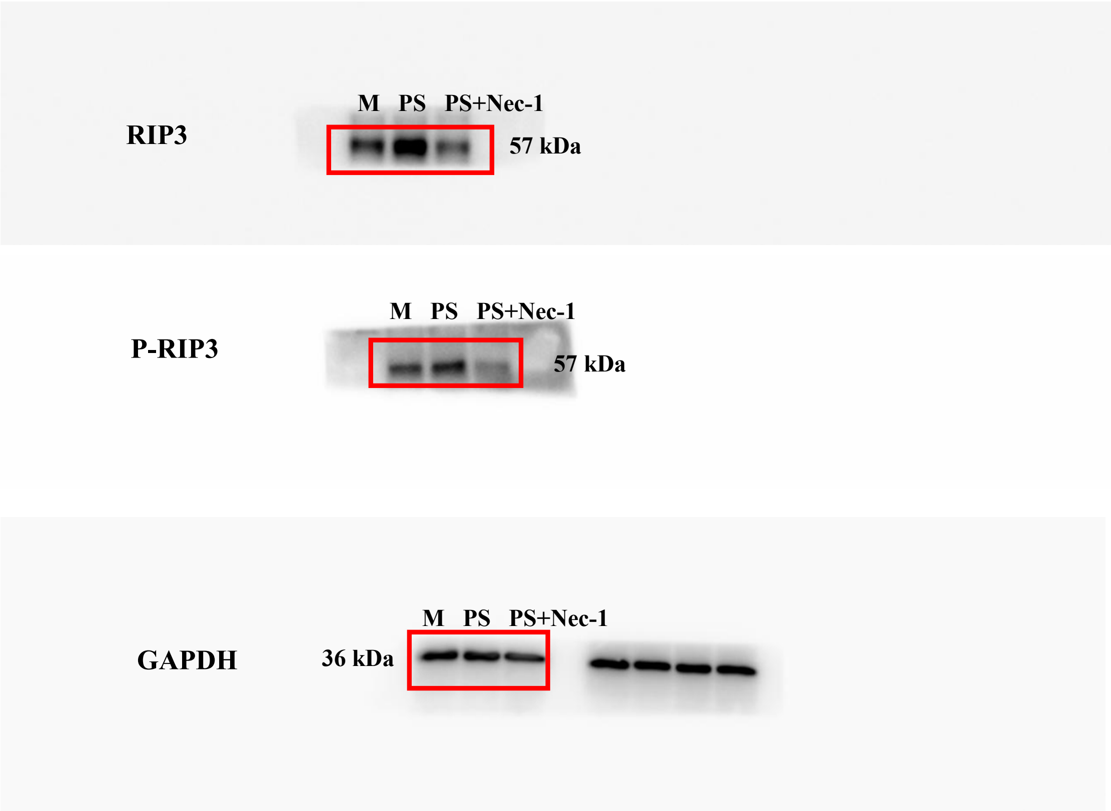
RIP3
M PS PS+Nec-1
57 kDa
P-RIP3
M PS PS+Nec-1
57 kDa
GAPDH
36 kDa
M PS PS+Nec-1

Figure 7(D)

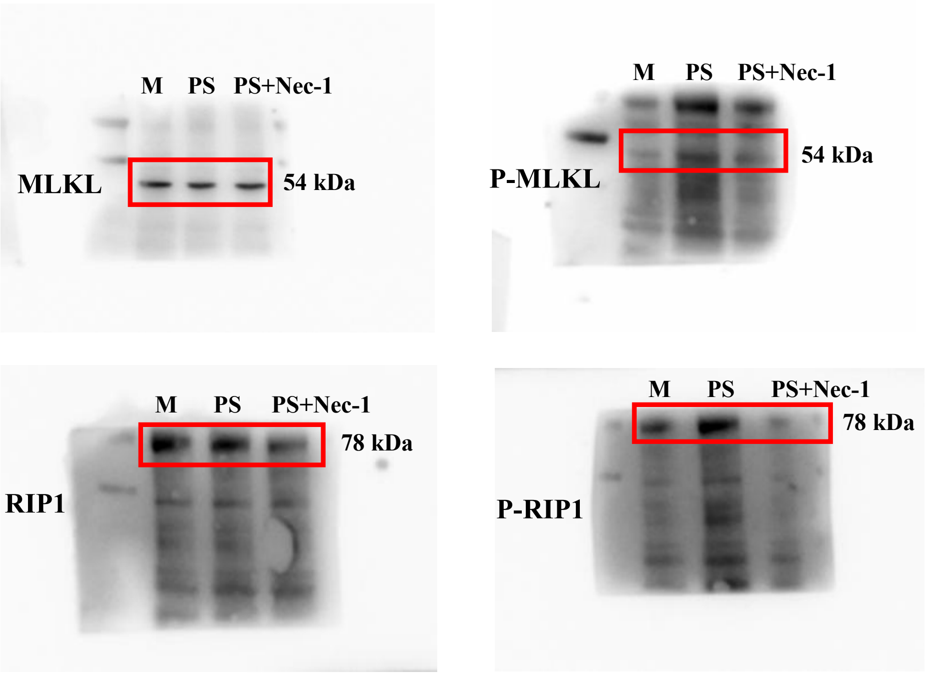
M PS PS+Nec-1
MLKL
54 kDa
M PS PS+Nec-1
P-MLKL
54 kDa
M PS PS+Nec-1
RIP1
78 kDa
M PS PS+Nec-1
P-RIP1
78 kDa

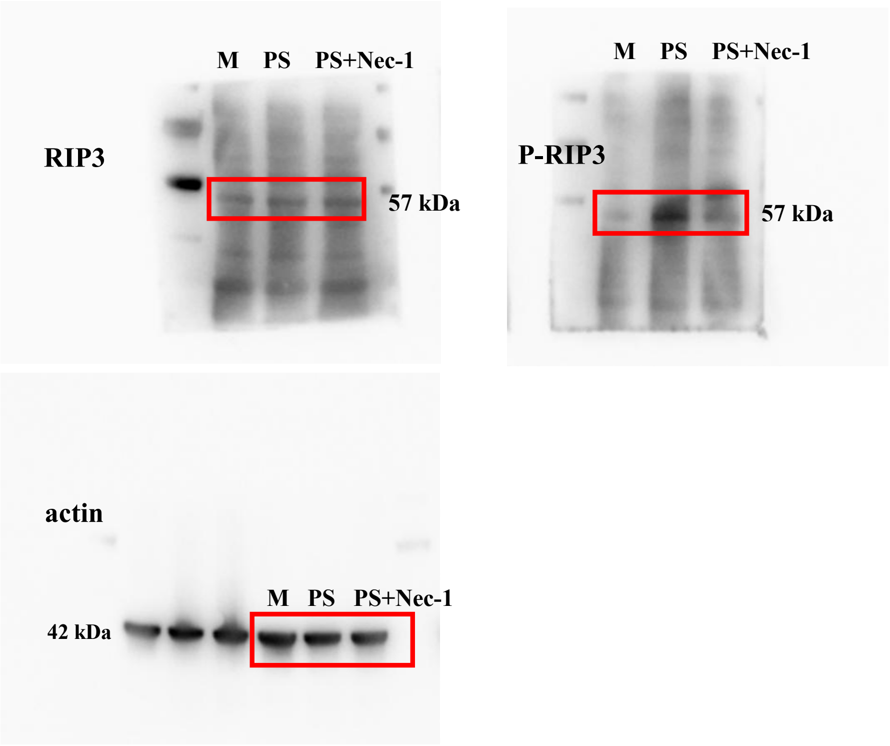
M PS PS+Nec-1
RIP3
57 kDa
M PS PS+Nec-1
P-RIP3
57 kDa
actin
M PS PS+Nec-1
42 kDa
