# Supplementary material for: Acute exposure to polystyrene nanoparticles promotes liver injury by inducing mitochondrial ROS-dependent necroptosis and augmenting macrophage-hepatocyte crosstalk
Source: Part Fibre Toxicol. 2024 Apr 12;21:20. doi: 10.1186/s12989-024-00578-6 (PMC11010371; doi:10.1186/s12989-024-00578-6)
Supplement: Supplementary file 2 — Supplementary Material 2 [file 12989_2024_578_MOESM2_ESM.docx]

**Additional file:**

**S. Tab. 1 Characterization of PS dispersion in DI water and DMEM.**

|  | PSNPs (20 nm) | | PSNPs (100 nm) | | PSMPs (1 μm) | |
| --- | --- | --- | --- | --- | --- | --- |
|  | DI water | DMEM | DI water | DMEM | DI water | DMEM |
| Size (nm) | 25.85±0.42 | 30.14±0.76 | 105.87±1.31 | 103.77±0.52 | 1207.33±33.88 | 1274.67±29.39 |
| PDI | 0.26±0.01 | 0.22±0.03 | 0.01±0.01 | 0.01±0.01 | 0.12±0.77 | 0.09±0.05 |
| Zeta potential (mV) | -33.7±1.47 | -22.8±0.99 | -40.1±0.22 | -24.3±0.29 | -22.57±0.21 | -2.43±0.25 |


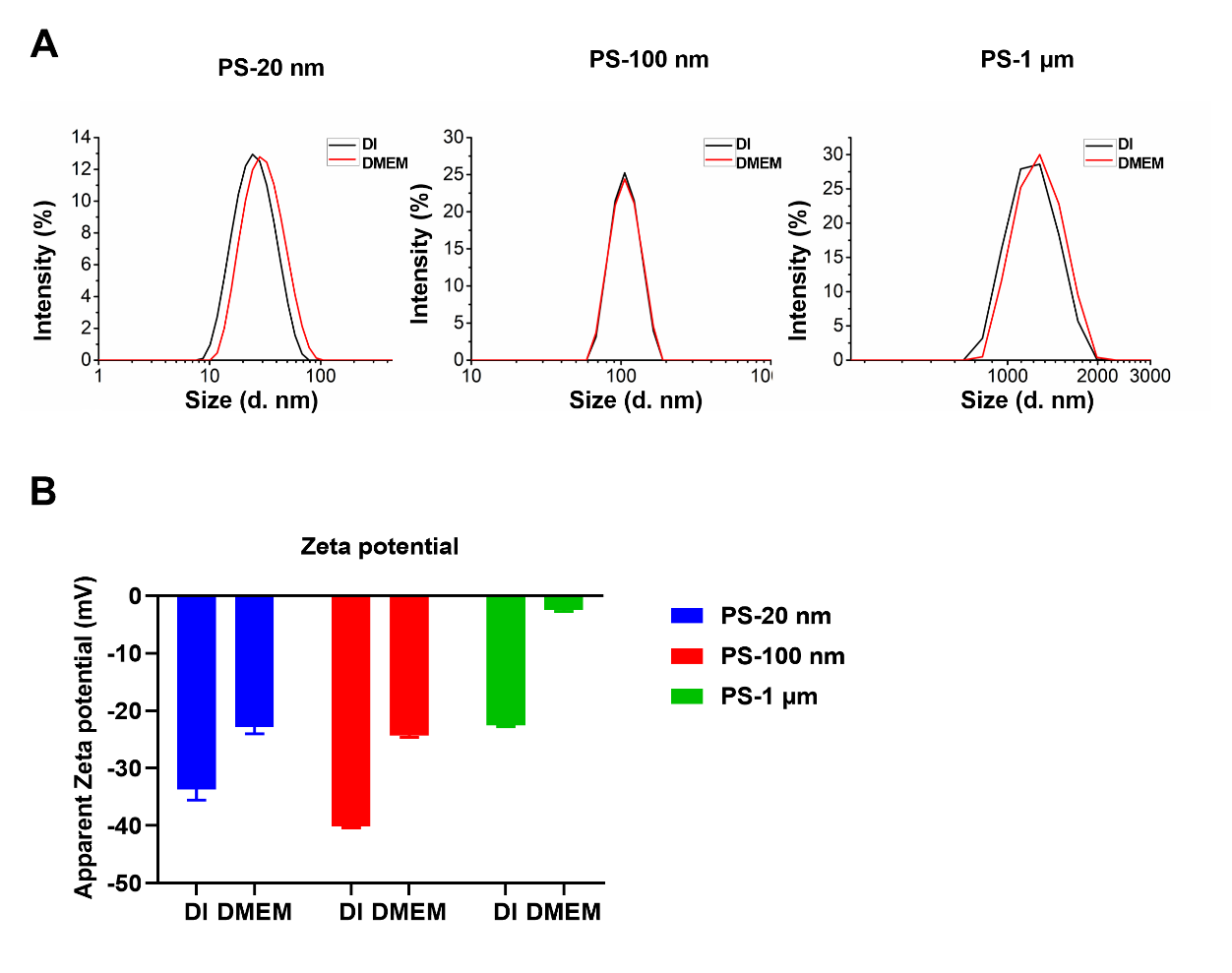


**S. Fig. 1** Characterization of 20 nm, 100 nm and 1 μm PS particles. (A-B) Average size distribution and zeta potential in DI water and DMEM.


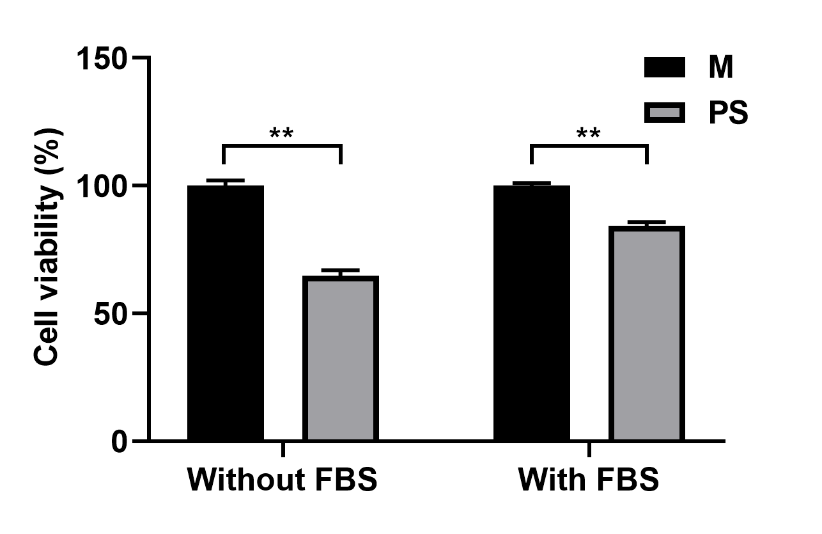


**S. Fig. 2** Effect of 20 nm PSNPs (50 μg/mL) on the viability of RAW 264.7 cells cultured with or without FBS for 4 h. *N.S., no significance. * P < 0.05, **s P < 0.01* 20 nm PSNPs at 50 μg/mL or 60 mg/kg were used in vitro or in vivo respectively.


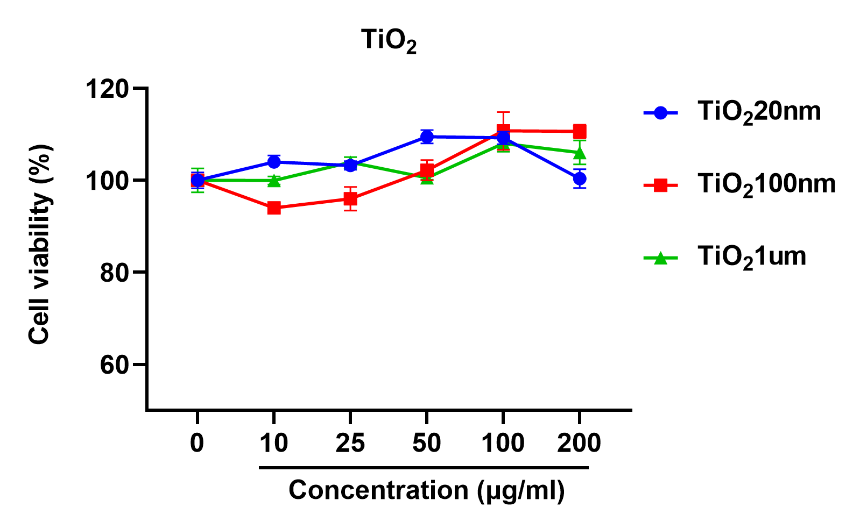


**S. Fig. 3** Comparison of the time and dose effects on the viability of RAW 264.7 cells by 20 nm, 100 nm and 1 μm TiO2. With the increase of concentration, different sizes of TiO2 had no obvious toxic effect on RAW 264.7 cells.


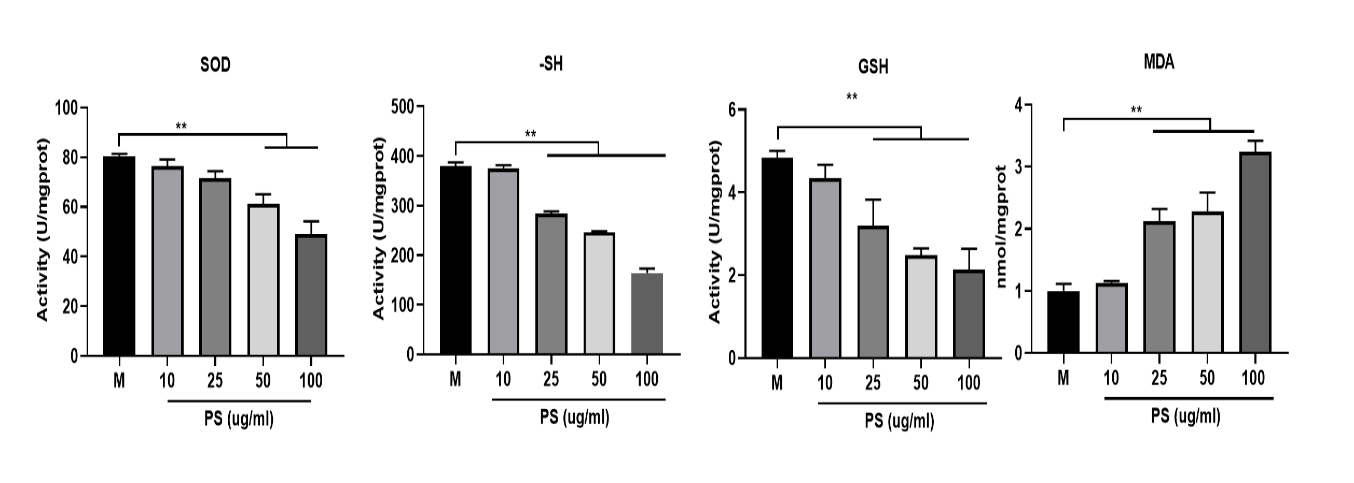


**S. Fig. 4** Levels of SOD, -SH, GSH and MDA in RAW 264.7 cells treated with 10-100 μg/mL PSNPs for 4 h. *N.S., no significance. * P < 0.05, **s P < 0.01* 20 nm PSNPs at 50 μg/mL or 60 mg/kg were used in vitro or in vivo respectively.

**
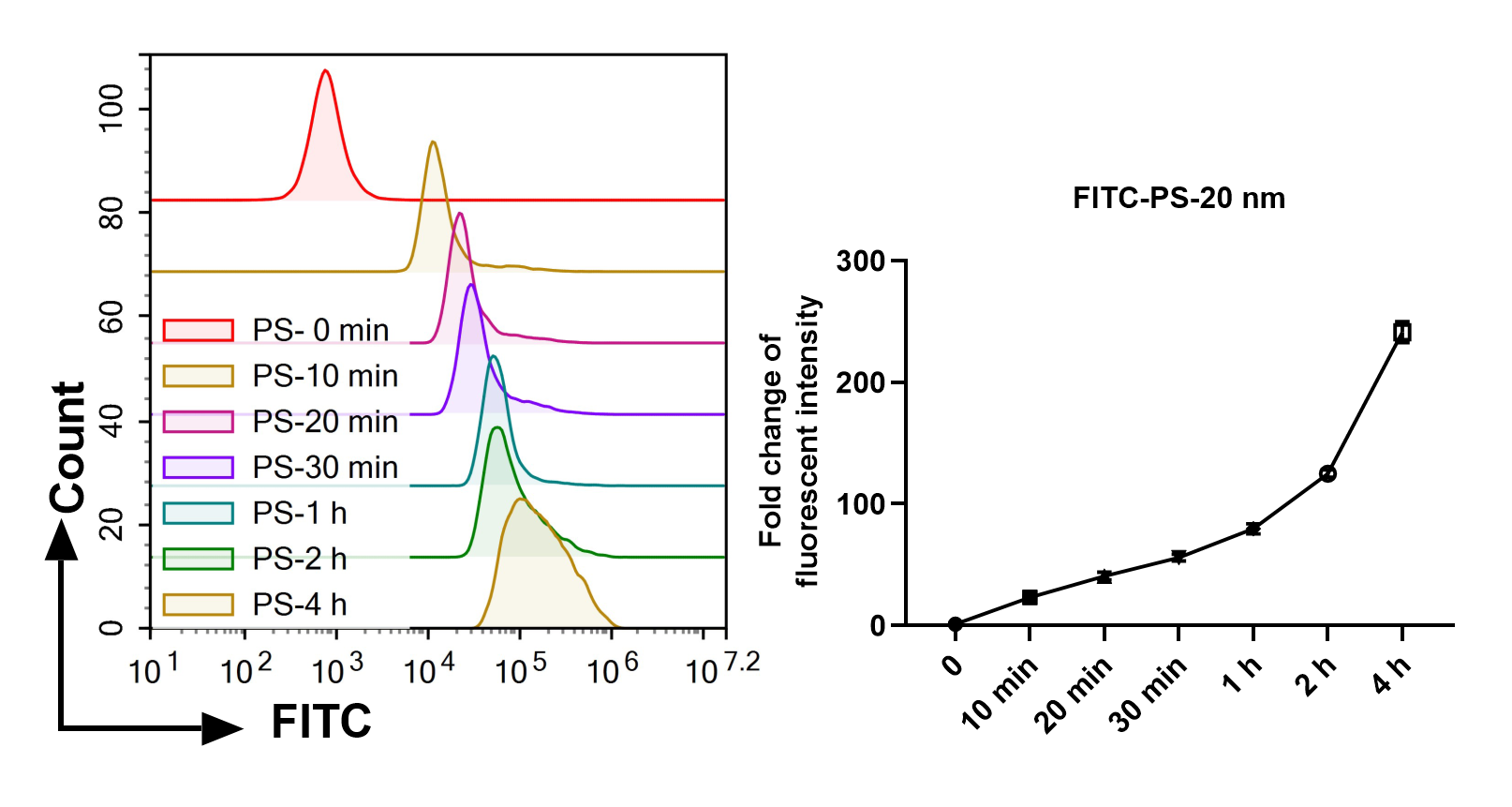
**

**S. Fig. 5** Detection of uptake of FITC-PS by RAW 264.7 cells at different incubation time by flow cytometry. With the extension of time, FITC-PS uptake increased.


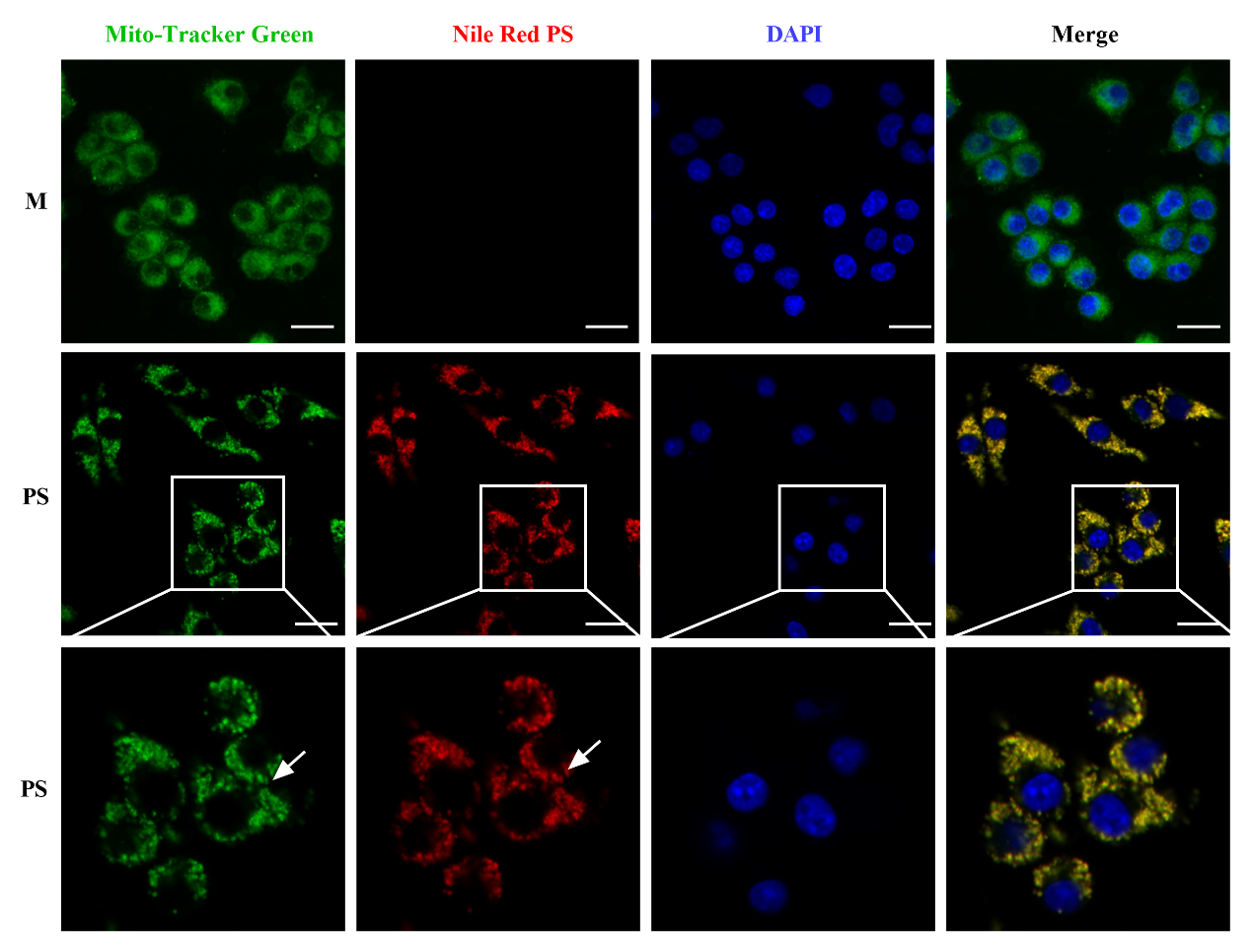


**S. Fig. 6** Mitochondrial location of Nile Red-labeled PS (50 μg/ml) with mitochondria (Mito-tracker green) in J774A.1 cells. It can be clearly seen that Red-labeled PS and Mito-tracker green are co-located. Scale bar 20 μm.


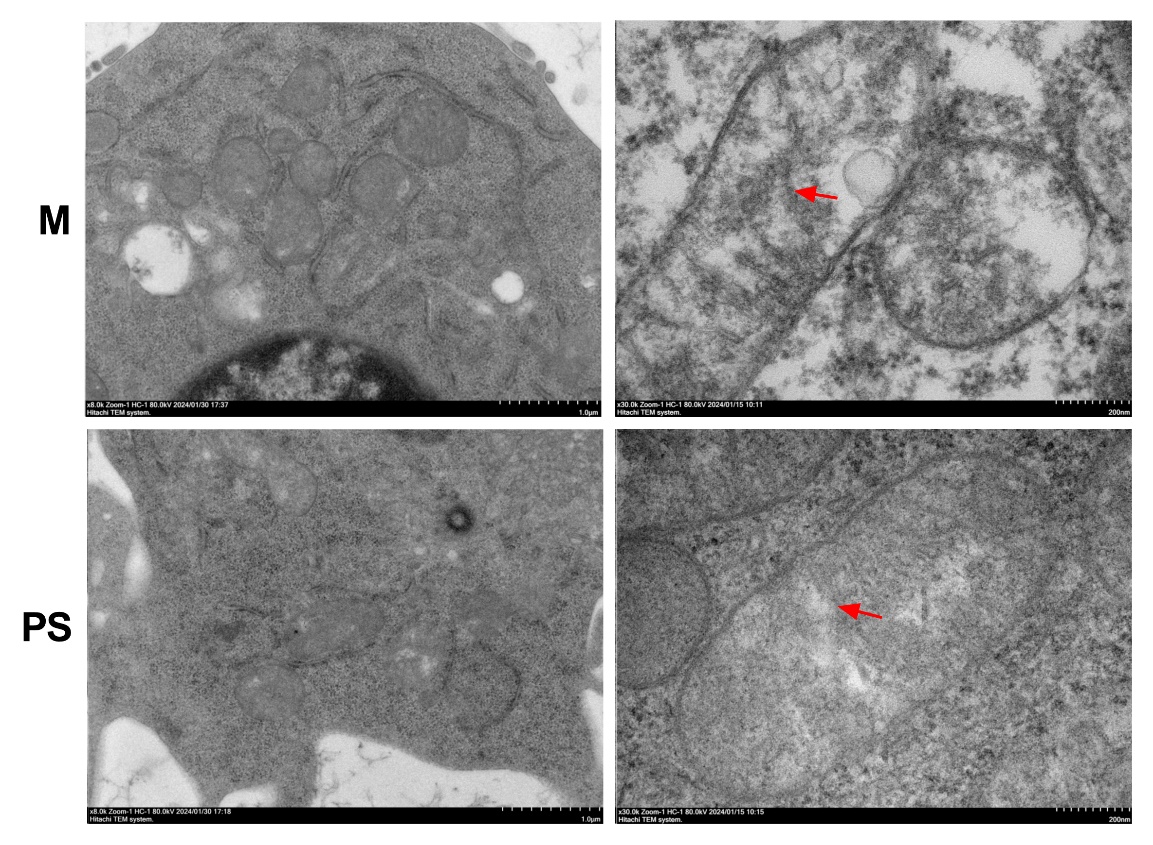


**S. Fig. 7** Microstructure of mitochondria was observed by TEM. The cell membrane of PS treatment group ruptured. Red arrow indicated the mitochondria with fractured and fuzzy cristae. Scale bar 1 μm and 200 nm, respectively.


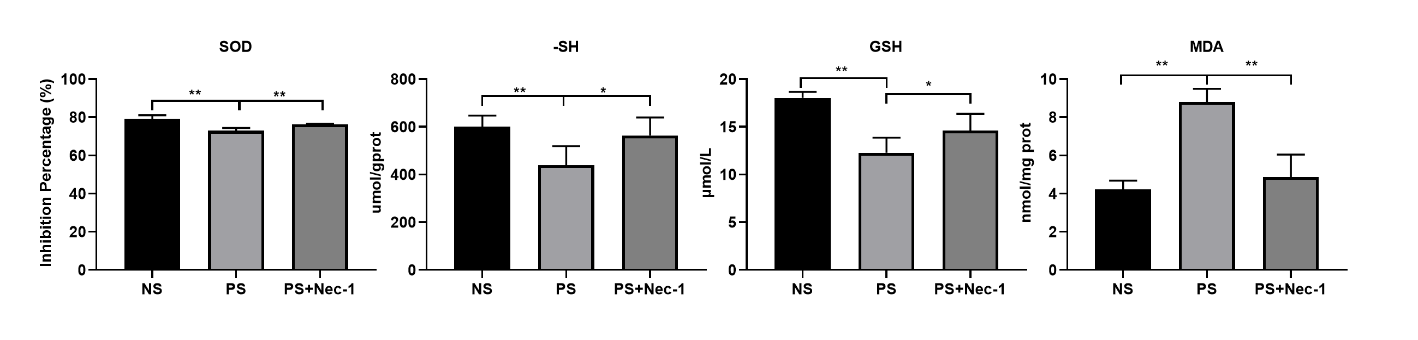


**S. Fig. 8** Levels of SOD, -SH, GSH and MDA in BALB/c mice treated with 60 mg/kg PSNPs for 24 h. *N.S., no significance. * P < 0.05, **s P < 0.01* 20 nm PSNPs at 50 μg/mL or 60 mg/kg were used in vitro or in vivo respectively.
